# Comprehensive analysis of a novel four-lncRNA signature as a prognostic biomarker for human gastric cancer

**DOI:** 10.18632/oncotarget.20496

**Published:** 2017-08-24

**Authors:** Yan Miao, Jing Sui, Si-Yi Xu, Ge-Yu Liang, Yue-Pu Pu, Li-Hong Yin

**Affiliations:** ^1^ Key Laboratory of Environmental Medicine Engineering, Ministry of Education, School of Public Health, Southeast University, Nanjing, Jiangsu 210009, P.R. China

**Keywords:** lncRNA, GC, prognostic biomarker, overall survival, TCGA

## Abstract

Emerging evidence indicates that long non-coding RNAs (lncRNAs) play a crucial role in predicting survival for gastric cancer (GC) patients. This study aims to identify a lncRNA-related signature for evaluating the overall survival of 379 GC patients from The Cancer Genome Atlas (TCGA) database. The associations between survival outcome and the expression of lncRNAs were evaluated by the univariate and multivariate Cox proportional hazards regression analyses. Four lncRNAs (LINC01018, LOC553137, MIR4435-2HG, and TTTY14) were identified as significantly correlated with overall survival. These four lncRNAs were gathered as a single prognostic signature. There was a significant positive correlation between GC patients with low-risk scores and overall survival (P = 0.001). Further analysis suggested that the prognostic value of this four-lncRNA signature was independent in clinical features. Gene set enrichment analysis found that these four lncRNAs were correlated with several molecular pathways of the tumor. Our study indicates that this novel lncRNA expression signature may be a useful biomarker of the prognosis for GC patients, based on bioinformatics analysis.

## INTRODUCTION

Gastric cancer (GC) belongs to one of the most frequently diagnosed cancer in the world with both high mortality and incidence. According to the Global Cancer Statistics 2012, more than 7.2 million GC-related deaths and about 9.5 million new diagnosed cases occurred worldwide [1]. Moreover, GC ranked the second in both the most common incident cancer and the leading cause of cancer death in China, 2015 [2]. The poor prognosis of GC patients is a significant reflection of the fact that most GC cases are diagnosed at advanced stages [3]. The detection of GC in an early stage, effective prediction of outcomes before treatment, and development of novel therapeutic targets are effective strategies to improve the prognosis of GC. Therefore, the identification of new biomarkers related to prognosis is essential for improving outcomes in GC patients.

Long non-coding RNAs (lncRNAs), greater than 200 nucleotides that have no protein-coding potential. LncRNAs have been widely identified in various diseases, including cancers. According to the recent evidence, lncRNAs can regulate different processes of gene expression by sequestering and binding them [4]. LncRNAs play critical roles in a variety of mechanisms, including cell development and differentiation [5], cell growth arrest and apoptosis [6], and X chromosome inactivation [7].

A series of lncRNAs have been discovered and confirmed as tumor suppressors or oncogenes. For example, MEG3 played as a tumor suppressor through the activation of p53 [8], and H19 performed as an oncogene in GC and colon cancer [9, 10]. Due to the contributions in the development and progression of cancer, lncRNAs were regarded as possible biomarkers for early diagnosis and prognosis. Till now, lncRNAs acted as biomarkers for diagnosis in GC have been reported in many studies. However, limited research reported the use of lncRNAs, especially lncRNA signature as biomarkers for Overall Survival (OS) in GC.

The object of this study aims to identify a novel lncRNA signature for GC prognosis through the data mining in The Cancer Genome Atlas (TCGA) (http://cancergenome.nih.gov). By performing a comprehensive lncRNA expression profile analysis, we identified a lncRNA signature in GC with four lncRNAs (LINC01018, LOC553137, MIR4435-2HG, and TTTY14), as a new candidate indicator with the potential to predict the OS in GC patients.

## RESULTS

### Patient characteristics

There were 379 GC patients and 35 normal controls included in the present study obtained from TCGA database. After the initial screening TNM stage, the GC patients were divided into four groups: stage I-, stage II-, stage III- and stage IV-group. The clinical features were summarized in Table 1. The mean ± standard deviation (STDEV) age for all patients was 65.189 ± 10.694. During the follow-up (mean ± STDEV: 599.800 ± 541.537 days), 151 of 379 (39.842%) patients died. Information on outcomes of first course treatment for 328 patients was available, including 231 (70.427%) achieved complete remission (CR), 6 (1.829%) partial remission (PR), 27 (8.232%) stable disease (SD), and 64 (19.512%) progressive disease (PD).

**Table 1:** The predictive values of related clinical features and risk score

Variables		Patient N=379
Race	White	230
	Black	11
	Asian	84
Gender	Female	136
	Male	238
Age	<=65	172
	>65	198
Tumor stage	I	53
	II	119
	III	163
	IV	39
T stage	T1	18
	T2	78
	T3	172
	T4	106
N stage	N0	117
	N1	98
	N2	75
	N3	78
M stage	M0	336
	M1	24
Histologic grade	G1	7
	G2	130
	G3	229
neoplasm subdivision	gastroesophageal junction	38
	cardia/proximal	50
	fundus/body	130
	antrum/distal	142
Primary therapy outcome	CR	231
	PR	6
	SD	27
	PD	64
Radiotherapy	NO	283
	YES	64
Targeted molecular	NO	187
	YES	157
Anti-reflux	NO	140
	YES	35
Family history	NO	283
	YES	18
HP infection	NO	144
	YES	20
Neoplasm cancer	Tumor free	221
	With tumor	125
Residual tumor	R0	308
	R1+R2	31

CR: complete remission; PR: partial remission; SD: stable disease, PD: progressive disease.

### Identification of differentially expressed lncRNAs

**Figure 1:**
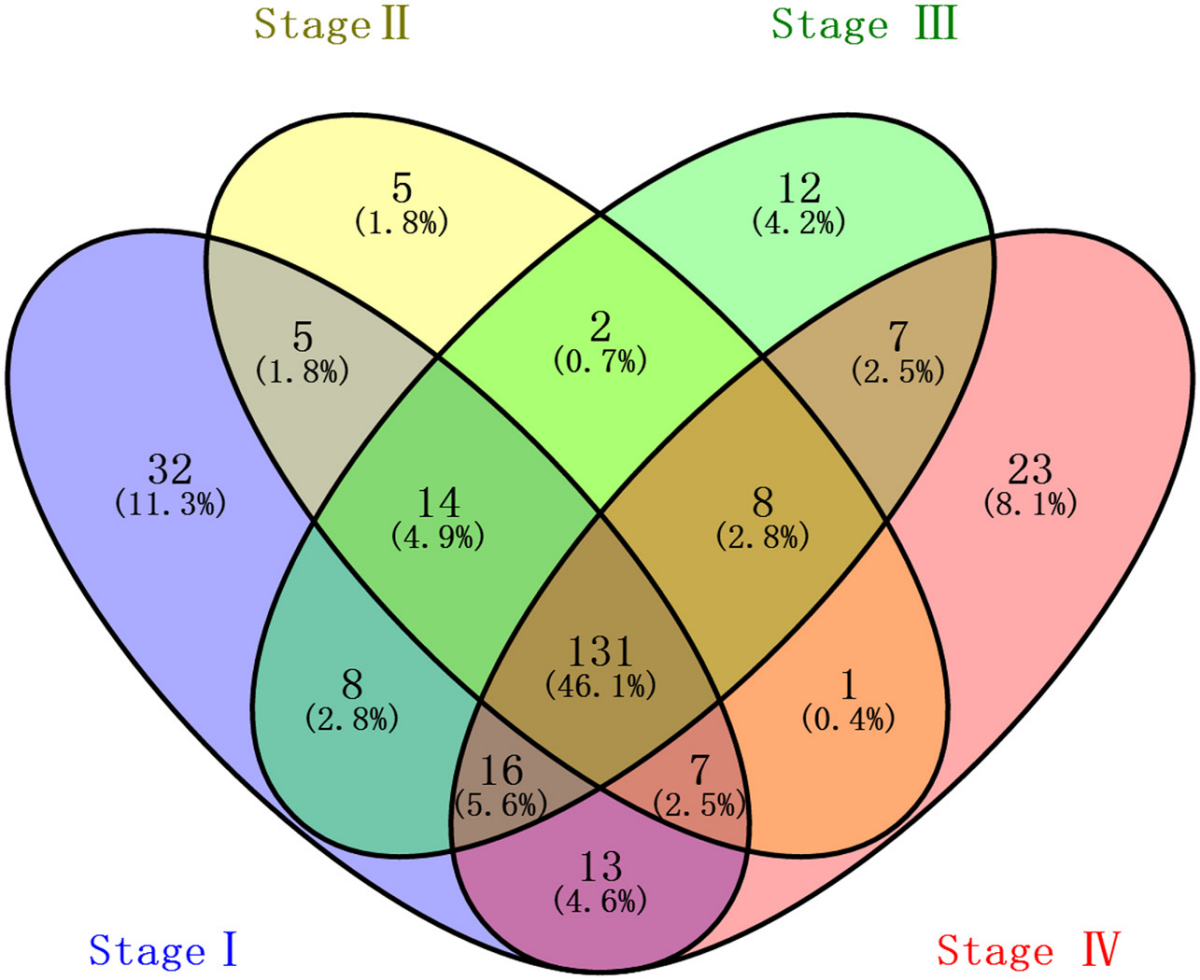
Venn diagram analysis of differentially expressed lncRNAs in gastric cancer Each oval represents a group. The brown intersection in the middle represents RNAs, which are consistently and significantly differentially expressed in four groups.

**Figure 2:**
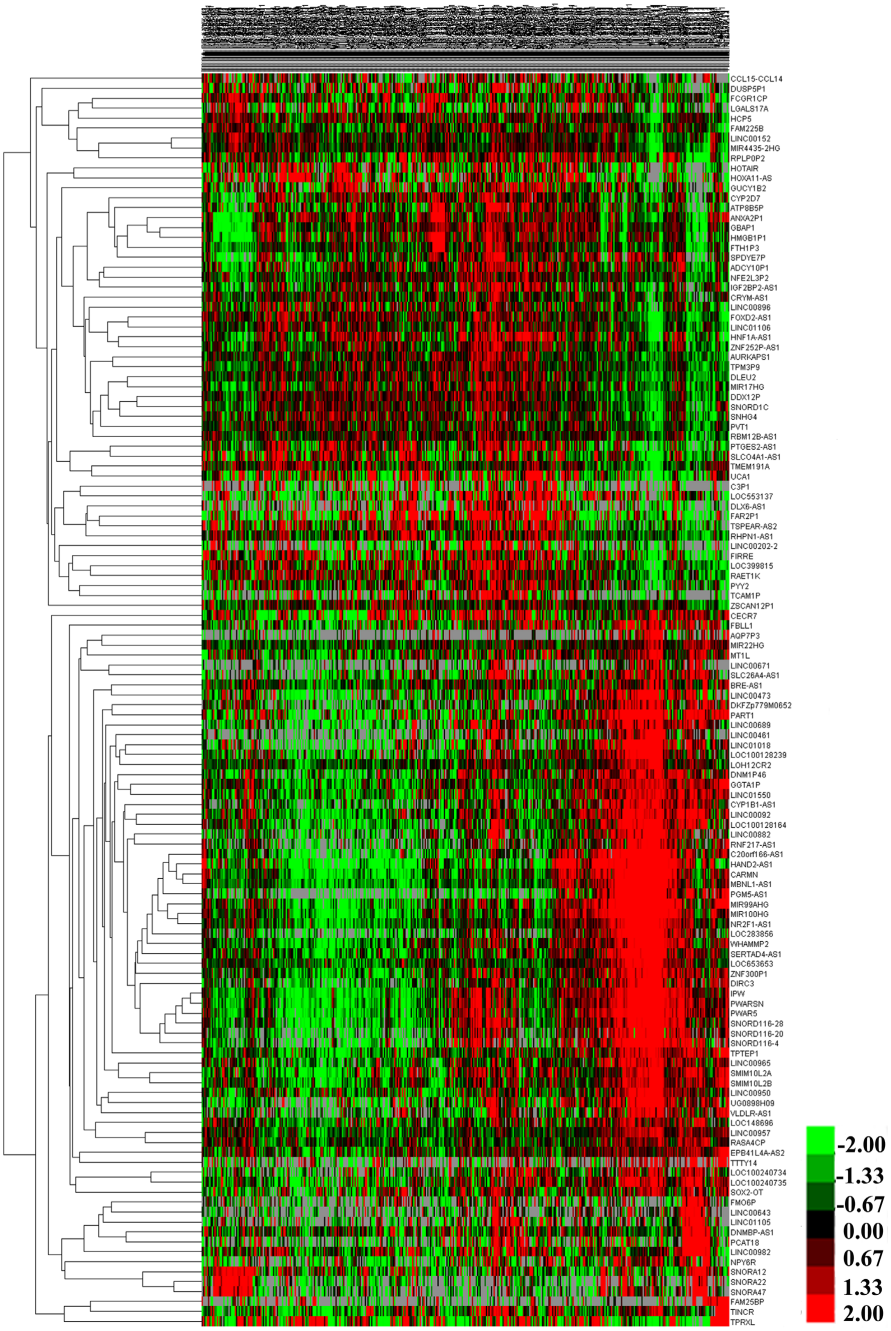
The differential expression of intersected lncRNAs in gastric cancer A heatmap is showing the differentially expressed RNAs.

1081 lncRNAs were identified from initially performed differential expression analysis from the TCGA database in GC. Fold change >2 and P value <0.05 were set up to be origins to identify significantly differentially expressed lncRNAs. Then we obtained 226 differentially expressed lncRNAs between stages I GC and adjacent normal gastric tissue, 173 differentially expressed lncRNAs between stages II GC and adjacent normal gastric tissue, 198 differentially expressed lncRNAs between stages III GC and adjacent normal gastric tissue, and 206 differentially expressed lncRNAs between stages IV GC and adjacent normal gastric tissue (fold change > 2, P value < 0.05). When we combined these four groups of differentially expressed lncRNAs together, 131 differentially expressed lncRNAs showed consistently differential expression (Figure 1 and Figure 2).

### Identification of lncRNA significantly associated with OS and prognostic signature construction

**Figure 3:**
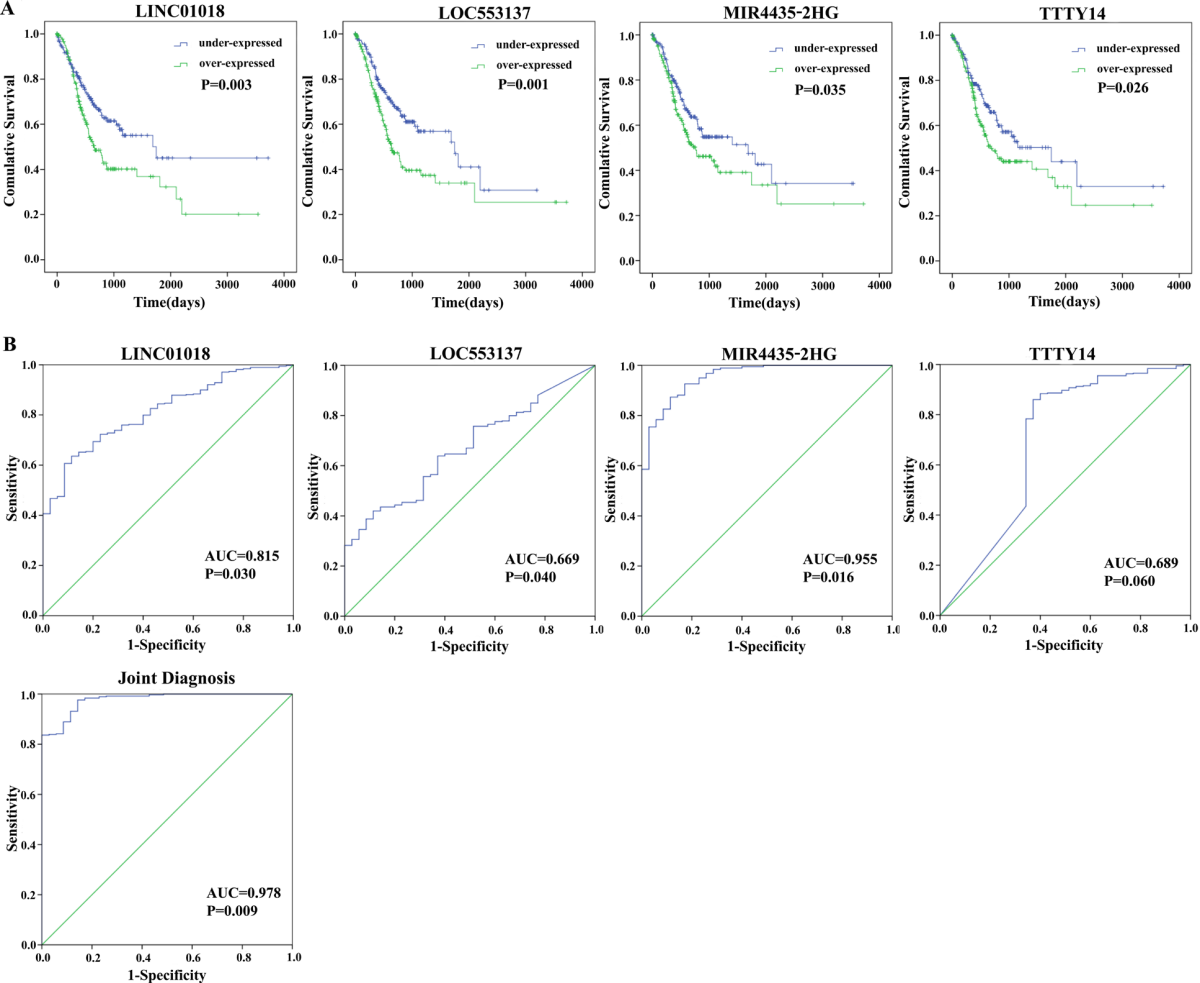
Four differentially expressed lncRNAs (LINC01018, LOC553137, MIR4435-2HG, and TTTY14) **(A)** Kaplan-Meier curves showing the relationship between the four lncRNAs and overall survival. The cases were divided into under- and over-expression groups by the mean lncRNAs level; **(B)** ROC curves of the four lncRNAs to distinguish gastric cancer tissue from adjacent normal tissues.

By subjecting differentially expressed lncRNAs expression data in 379 patients from TCGA database to the univariate Cox regression model, a total of 23 lncRNAs were identified as candidate biomarkers significantly associated with OS (P-value < 0.05) (Table 2). Multivariate Cox regression analysis was performed to take into account for the interrelated relationship among 23 lncRNAs and identified four lncRNAs (LINC01018, LOC553137, MIR4435-2HG, and TTTY14) as independent biomarkers for OS in GC patients (P < 0.05) (Table 3 and Figure 3).

**Table 2:** Prognostic value of the differentially expressed lncRNAs by univariate cox regression analysis

LncRNA	Estimate	StdErr	ChiSq	P	HR( 95%CI)
CARMN	0.449	0.166	7.271	0.007*	1.566(1.130-2.170)
CYP2D7	-0.345	0.167	4.279	0.039*	0.708(0.511-0.982)
DNM1P46	0.439	0.168	6.851	0.009*	1.551(1.117-2.154)
HAND2-AS1	0.378	0.167	5.132	0.023*	1.459(1.052-2.024)
LINC00461	0.349	0.166	4.404	0.036*	1.417(1.023-1.962)
LINC00473	0.348	0.166	4.375	0.036*	1.416(1.022-1.963)
LINC00908	0.395	0.167	5.598	0.018*	1.484(1.070-2.059)
LINC00965	0.357	0.168	4.529	0.033*	1.428(1.029-1.984)
LINC01018	0.491	0.169	8.481	0.004*	1.634(1.174-2.275)
LOC100128239	0.368	0.167	4.876	0.027*	1.445(1.042-2.003)
LOC553137	0.541	0.168	10.386	0.001*	1.718(1.236-2.387)
MIR100HG	0.381	0.166	5.247	0.022*	1.464(1.057-2.028)
MIR4435-2HG	0.348	0.166	4.390	0.036*	1.417(1.023-1.962)
MIR99AHG	0.530	0.168	9.967	0.002*	1.699(1.223-2.361)
NR2F1-AS1	0.471	0.167	7.937	0.005*	1.601(1.154-2.222)
PWAR5	0.049	0.167	6.004	0.014*	1.505(1.085-2.086)
RNF217-AS1	0.399	0.167	5.675	0.017*	1.490(1.073-2.068)
SMIM10L2A	0.334	0.167	3.981	0.046*	1.397(1.006-1.939)
SMIM10L2B	0.524	0.170	9.507	0.002*	1.689(1.210-2.357)
SNORD116-20	0.366	0.168	4.752	0.029*	1.441(1.038-2.002)
TTTY14	0.370	0.167	4.901	0.027*	1.447(1.043-2.008)
VLDLR-AS1	0.473	0.167	7.983	0.005*	1.605(1.156-2.228)
WHAMMP2	0.515	0.168	9.443	0.002*	1.674(1.205-2.325)

HR: hazard ratio; CI: confidence interval; *: P<0.05.

**Table 3:** Prognostic value of the differentially expressed lncRNAs by multivariate Cox regression analysis

LncRNA	Estimate	StdErr	ChiSq	P	HR( 95%CI)
LINC01018	0.455	0.170	7.164	0.007*	1.577(1.130-2.201)
LOC553137	0.483	0.169	8.147	0.004*	1.621(1.163-2.258)
MIR4435-2HG	0.361	0.167	4.699	0.030*	1.435(1.035-1.990)
TTTY14	0.389	0.167	5.393	0.020*	1.475(1.062-2.047)

HR: hazard ratio; CI: confidence interval; *: P<0.05.

We performed univariate Cox regression analysis to identify the four lncRNAs within each subclass of clinical features as follow: TNM stage, T stage, M stage, and N stage. Table 4 presented the HR for the association of these four lncRNAs with OS in each category.

**Table 4:** lncRNAs associated with prognosis in different clinical subclasses

lncRNA	Tumor stage I/II HR(95%CI)	Tumor stage III/IV HR(95%CI)	T I/II HR(95%CI)	T III/IV HR(95%CI)	N I/II HR(95%CI)	N III/IV HR(95%CI)
LINC01018	-	1.380(1.044-1.823)	-	1.361(1.073-1.726)	-	1.269(1.002-1.608)
LOC553137	-	1.381(1.045-1.824)	-	1.320(1.040-1.674)	-	1.315(1.037-1.668)
MIR4435-2HG	1.365(1.011-1.843)	-	-	1.370(1.080-1.738)	-	-
TTTY14	-	-	-	-	-	-

Afterwards, the risk score for predicting the OS was constructed with the formula: Risk score = exp_LINC01018_*(0.455) + exp_LOC553137_*(0.483) + exp_MIR4435-2HG_*(0.361) + exp_TTTY14_*(0.389).

**Figure 4:**
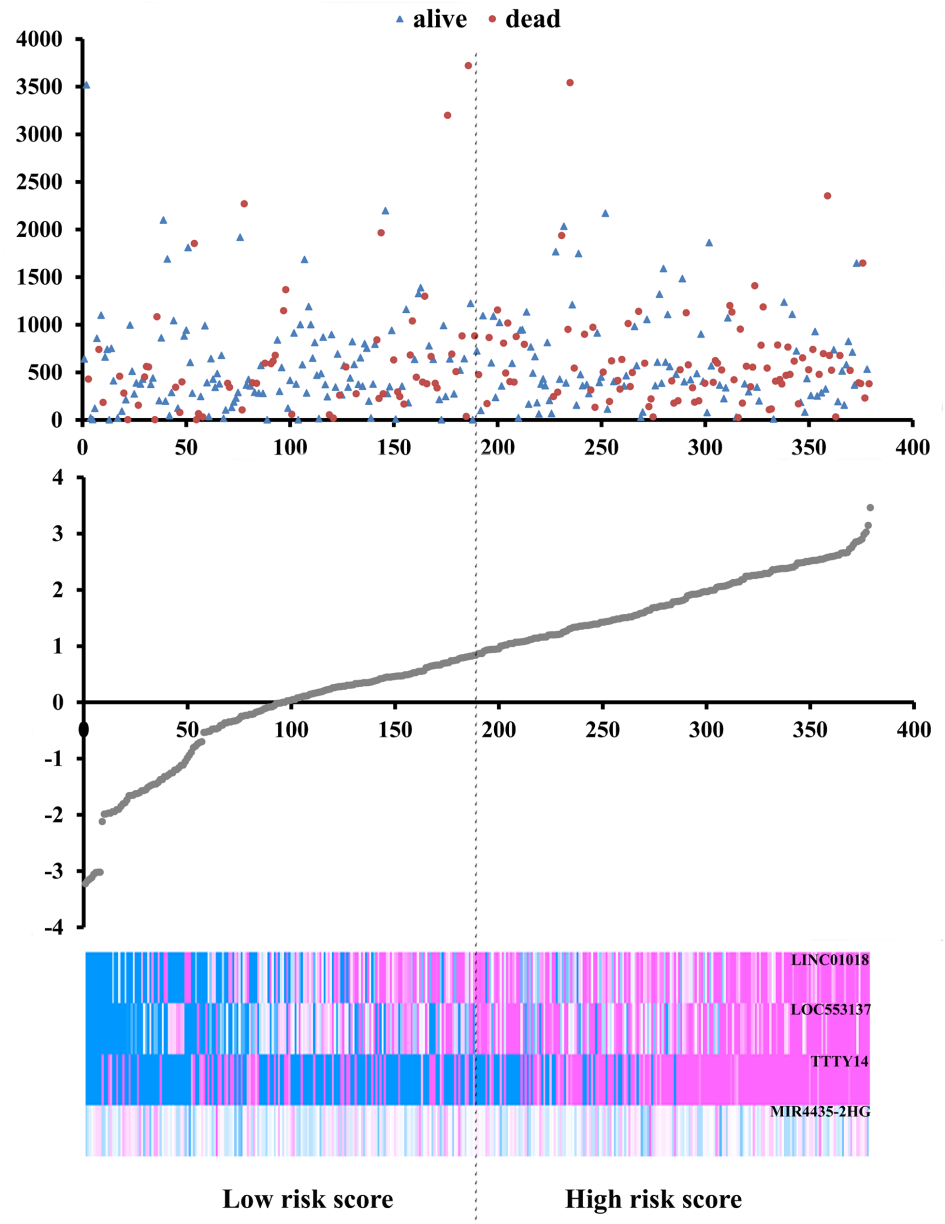
Risk score analysis of the differentially expressed lncRNA signature of gastric cancer Survival status and duration of cases (Top); risk score of lncRNA signature (Middle); low and high score groups for the four lncRNAs (Bottom).

**Figure 5:**
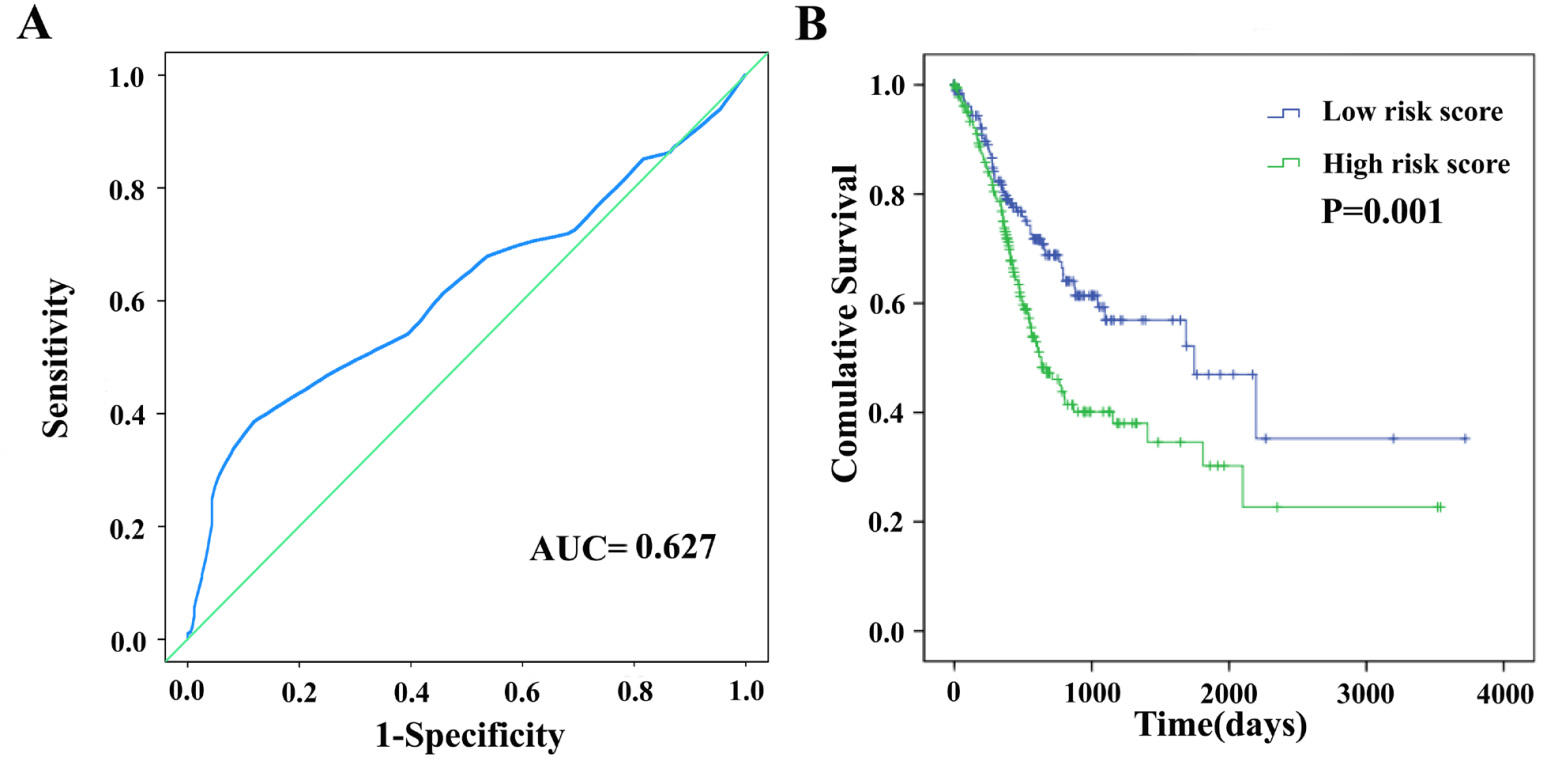
The four differentially expressed lncRNA signature of gastric cancer for the outcome **(A)** The risk score is shown by the time-dependent ROC curve for predicting 5-year survival. **(B)** The Kaplan-Meier test of the risk score for the overall survival.

Based on the risk score model mentioned above, GC patients were classified as low- or high-risk patients using the median risk score as the cutoff value, which divided into the low-risk group (n = 190) and high-risk group (n = 189) (Figure 4). The risk score could largely predict the 5-year survival of GC patients, as the area under ROC curve (AUC) was 0.627 (Figure 5A). Meanwhile, K-M curves confirmed that the survival time of patients in the low-risk group was 642.382 ± 533.037 days, predominantly longer than that of the high-risk group (561.128 ±517.423 days, P = 0.001, Figure 5B).

### The prognostic value of four-lncRNA signature is independent of other clinical features

Furthermore, to examine whether the prognostic value of the four-lncRNA signature is independent of other clinical features, the univariate and multivariate Cox proportional hazard regression analyses were performed to analyze with risk score and other clinical features, such as including race, age, gender, Tumor stage and T stage, as covariates in TCGA datasets.

**Figure 6:**
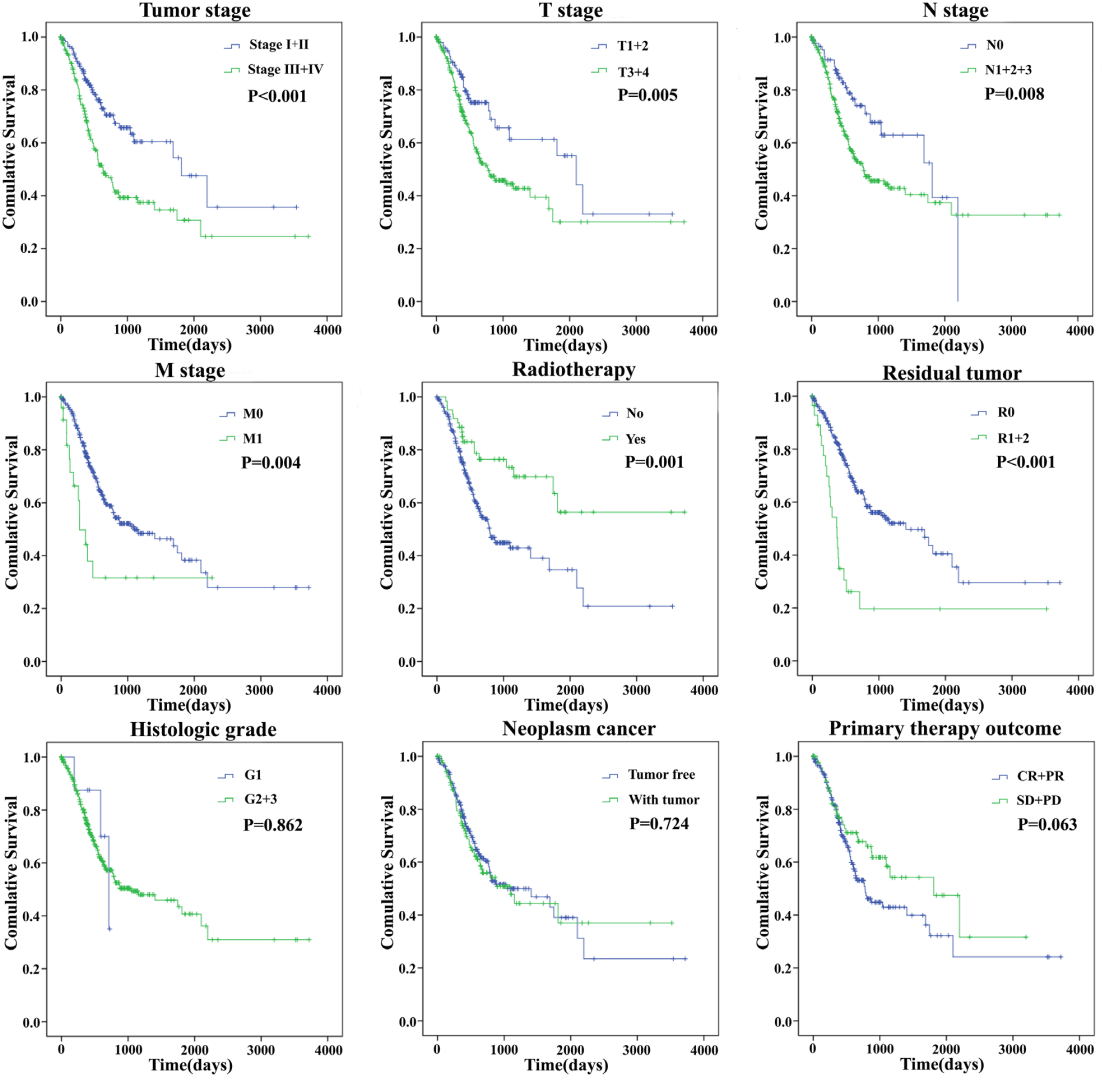
The prognostic value of different clinical features for overall survival of gastric cancer patients Kaplan-Meier curves of seven independent prognostic indicators. SD, stable disease; PD, progressive disease; CR, complete remission; PR, partial remission.

The univariate Cox proportional hazards regression showed that some features could predict poorer survival of GC, including age, Tumor stage, T stage, N stage, M stage, Primary therapy outcome, Radiotherapy, Residual tumor (Table 5). However, when analyzed by multivariate Cox proportional hazards regression test, only Residual tumor (P = 0.047) together with the risk score (P = 0.004), was an independent prognostic indicator of GC (Table 5). The K-M curves of the above clinical features are shown that Tumor stage (P < 0.001), T stage (P = 0.005), N stage (P = 0.008), M stage (P = 0.004), Residual tumor (P < 0.001), and Radiotherapy (P = 0.001) were associated with OS (Figure 6).

**Table 5:** The predictive values of related clinical features and risk score

Variables		Univariate analysis		Multivariate analysis	
		HR(95% CI)	P	HR(95% CI)	P
Race	White	1(reference)			
	Black	1.396(0.646-3.018)	0.397		
	Asian	0.839(0.534-1.318)	0.445		
Gender	Female	1(reference)			
	Male	1.239(0.874-1.756)	0.229		
Age	<=65	1(reference)			
	>65	1.621(1.161-2.263)	0.005*		
Tumor stage	I	1(reference)		1(reference)	
	II	1.505(0.779-2.905)	0.224	0.625(0.171-2.283)	0.946
	III	2.514(1.366-4.627)	0.003*	0.509(0.097-2.679)	0.956
	IV	4.016(1.996-8.079)	<0.001*	0.882(0.161-4.827)	0.435
T stage	T1	1(reference)		1(reference)	
	T2	7.405(1.005-54.535)	0.049*	2.633(0.337-20.601)	0.280
	T3	10.879(1.512-78.245)	0.018*	3.726(0.493-28.149)	0.375
	T4	10.799(1.485-78.519)	0.019*	3.747(0.488-28.756)	0.675
N stage	N0	1(reference)		1(reference)	
	N1	1.730(1.079-2.773)	0.023*	1.753(0.944-3.256)	0.075
	N2	1.777(1.062-2.974)	0.029*	1.560(0.794-3.066)	0.197
	N3	2.736(1.714-4.367)	<0.001*	2.475(1.328-4.616)	0.004*
M stage	M0	1(reference)		1(reference)	
	M1	2.279(1.287-4.042)	0.005*	1.034(0.411-2.604)	0.786
Histologic grade	G1	1(reference)		1(reference)	
	G2	1.004(0.312-3.234)	0.994	2.267(0.482-10.661)	0.395
	G3	1.167(0.369-3.692)	0.792	2.645(0.588-11.886)	0.267
neoplasm subdivision	gastroesophageal junction	1(reference)			
	cardia/proximal	1.467(0.732-2.938)	0.280		
	fundus/body	1.144(0.608-2.151)	0.677		
	antrum/distal	1.332(0.714-2.487)	0.368		
Primary therapy outcome	CR	1(reference)			
	PR	3.021(1.105-8.258)	0.031*		
	SD	0.829(0.441-1.559)	0.560		
	PD	0.651(0.404-1.048)	0.077		
Radiotherapy	NO	1(reference)			
	YES	0.427(0.255-0.715)	0.001*		
Targeted molecular	NO	1(reference)			
	YES	1.116(0.792-1.573)	0.530		
Anti-reflux	NO	1(reference)			
	YES	0.838(0.467-1.502)	0.553		
Family history	NO	1(reference)		1(reference)	
	YES	1.071(0.543-2.114)	0.842	1.057(0.492-2.271)	0.875
HP infection	NO	1(reference)			
	YES	0.463(0.168-1.281)	0.138		
Neoplasm cancer	Tumor free	1(reference)		1(reference)	
	With tumor	1.065(0.752-1.507)	0.724	1.243(0.819-1.887)	0.305
Residual tumor	R0	1(reference)		1(reference)	
	R1+R2	3.068(1.895-4.967)	<0.001*	1.876(1.009-3.487)	0.047*
Risk score	Low	1(reference)		1(reference)	
	High	1.753(1.257-2.444)	0.001*	1.889(1.228-2.905)	0.004*

HR: hazard ratio; CI: confidence interval; *: P<0.05.

**Figure 7:**
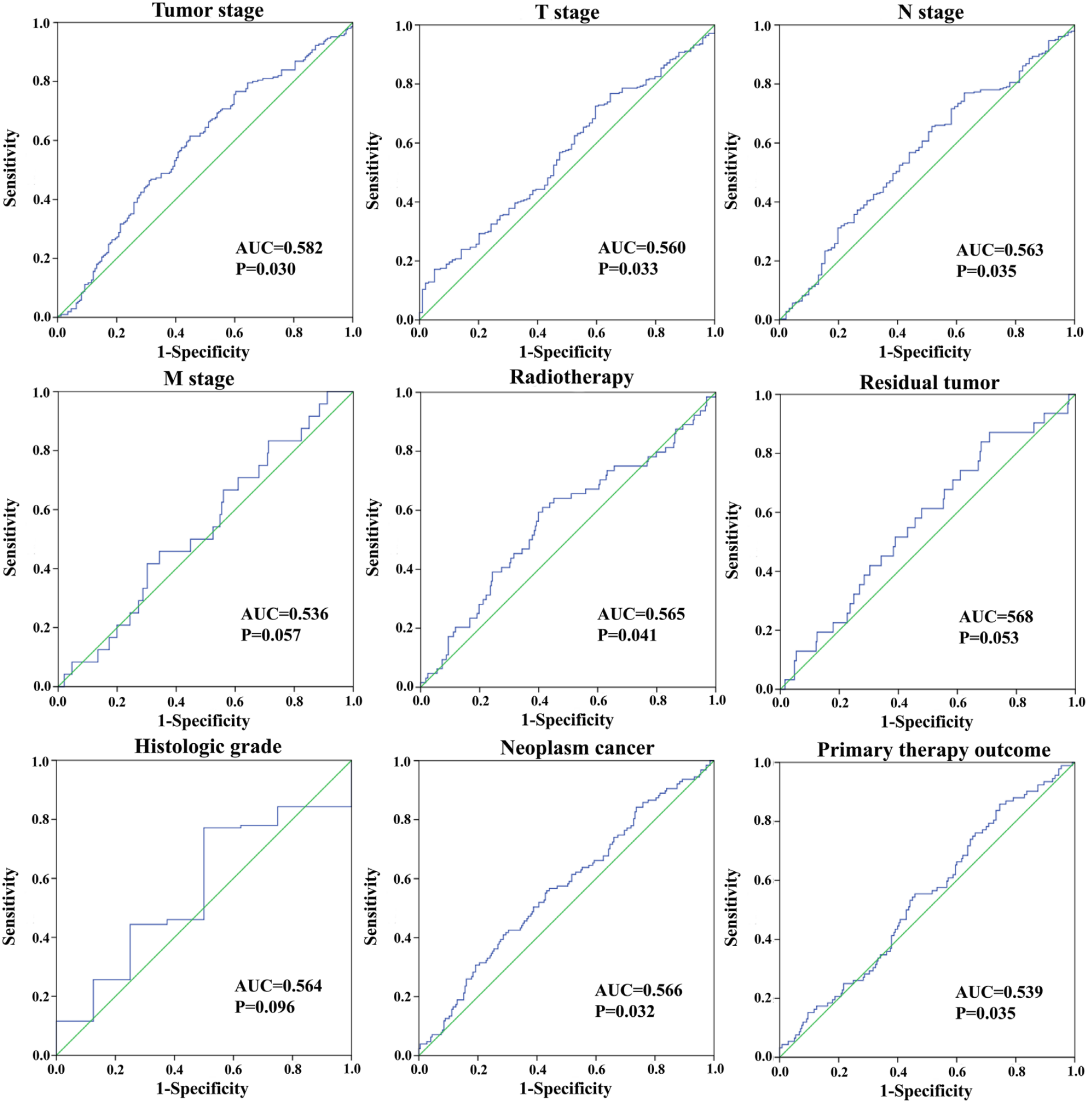
The predictive value of the risk score for clinical features ROC curve is predicting different clinical features.

**Figure 8:**
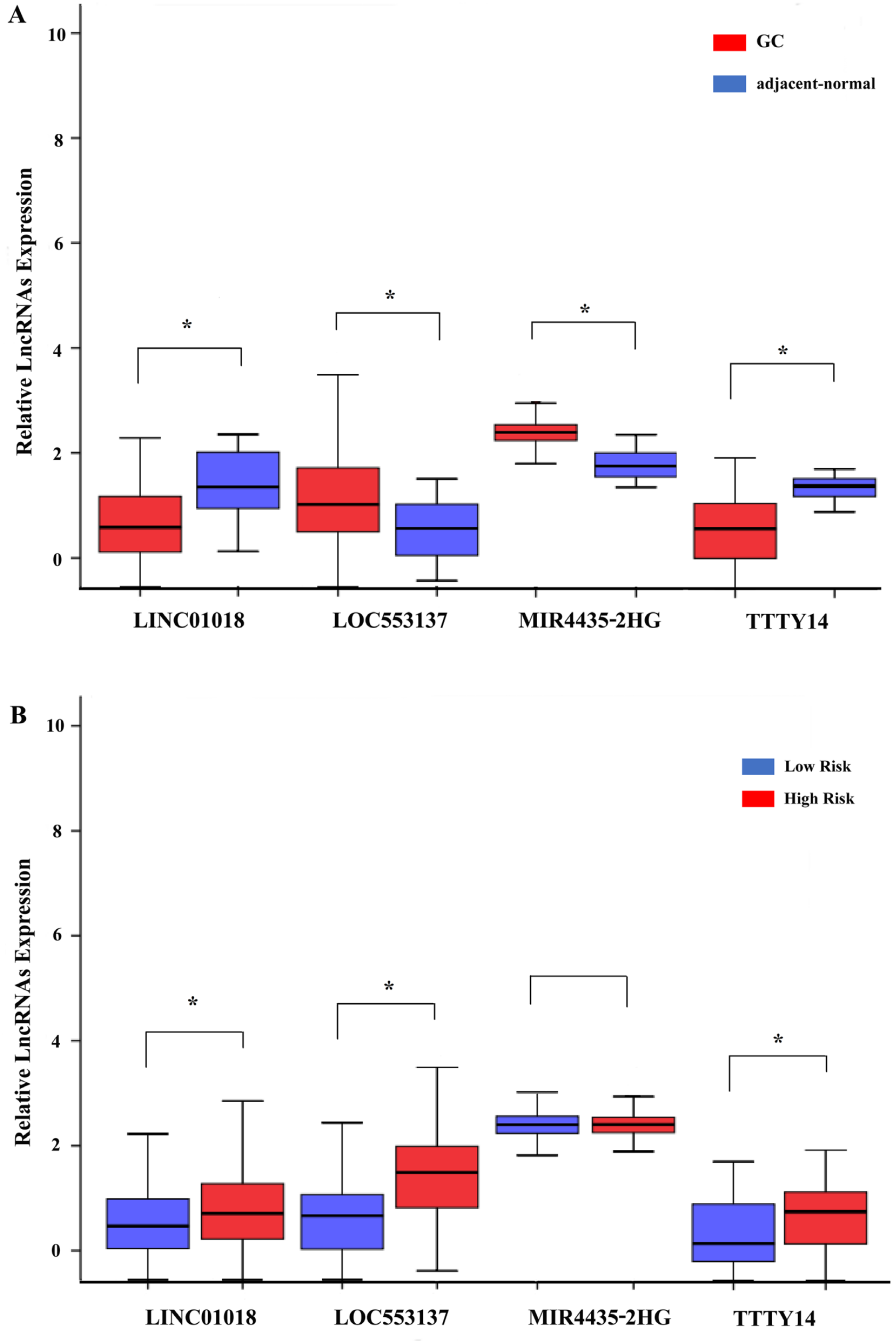
The expression level of the four lncRNAs (LINC01018, LOC553137, MIR4435-2HG, and TTTY14) **(A)** The expression level of lncRNAs between gastric cancer tissues and adjacent normal tissues; **(B)** The expression level of lncRNAs between the low-risk and high-risk groups. *P<0.05.

We assessed the relationship between the risk score based on the differentially expressed lncRNAs signature and various clinical features, and the risk score showed prognostic value for predicting the status (Figure 7). The expression pattern of these four differentially expressed lncRNAs in the GC and adjacent normal tissues, low- and high-score groups were shown in Figure 8.

### Functional assessment of the four lncRNAs

**Figure 9:**
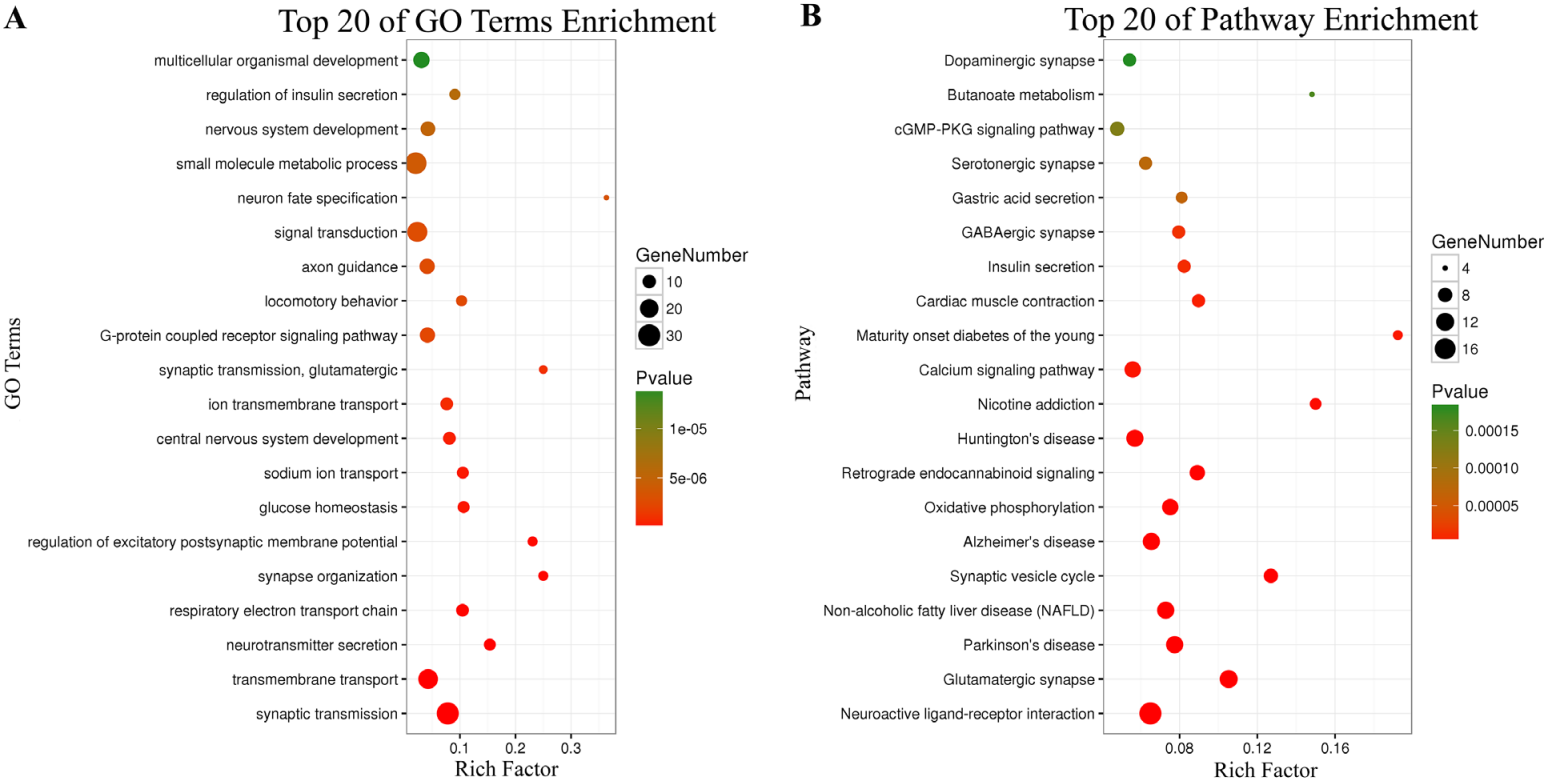
Top 20 enrichment of KEGG pathways and GO terms for co-expressed mRNAs

**Figure 10:**
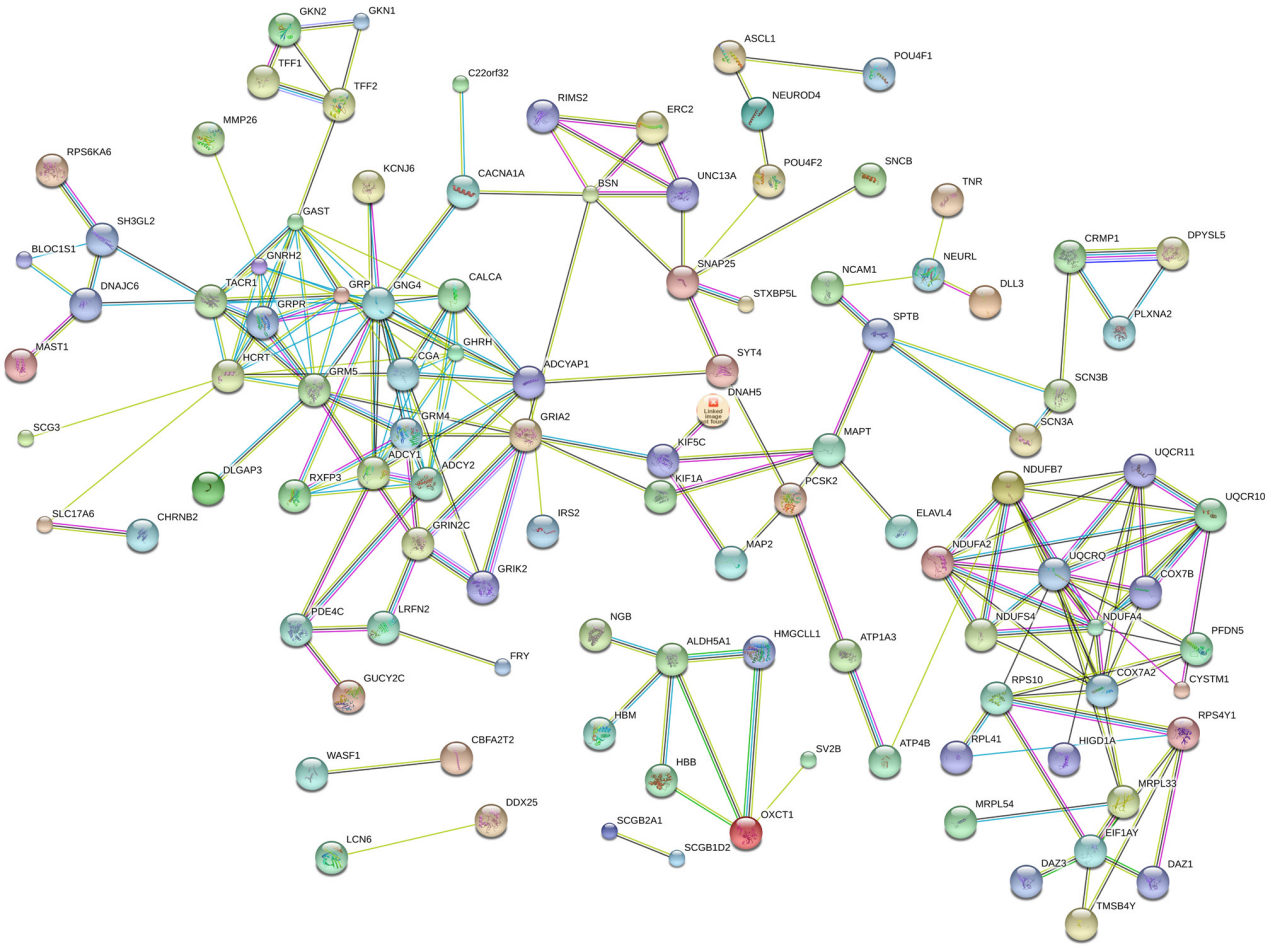
The map represents the protein-protein interaction network of co-expressed genes

There were 434 genes identified in TCGA database co-expressed with these four lncRNAs (LINC01018, LOC553137, MIR4435-2HG, and TTTY14) (|R| > 0.5) (Supplementary Table 1). It revealed enrichment of 240 GO Terms and 47 Pathways (P-value of <0.05 and an enrichment score of >1.5; Supplementary Table 2). It was found that the top GO biological process of co-expressed genes was synaptic transmission (GO: 0007268) and transmembrane transport (GO: 0055085) (Figure 9A). After the pathway analysis, the co-expressed genes were mainly enriched in Neuroactive ligand-receptor interaction and Glutamatergic synapse (Figure 9B). For the construction of the protein-protein interaction (PPI) network, there were 106 genes in the PPI network, which were regarded as hub genes (Figure 10).

## DISCUSSION

Gastric cancer (GC) is one of the deadliest solid tumors with the high global morbidity and mortality [11]. Although over several decades GC shows a slight decline in morbidity and mortality [12], it remains a significant clinical challenge owing to limited detection methods and poor prognosis [13]. The specific biomarkers for its early diagnosis, therapeutic process monitoring, and prognostic evaluation might increase survival rate. Accumulating evidence suggested that lncRNAs may play a major role in tumorigenesis, development, metastasis, the prognosis of GC [10, 14–17]. The recent large-scale genome analysis has revealed the molecular characteristics associated with GC OS [18]. However, most studies focused on miRNA, miRNAs, gene and protein expression [19–24]. With knowledge growing, the functional role of lncRNAs in tumorigenesis and development also represents a significant untapped resource for cancer prognosis.

In the present study, to identify lncRNAs significantly related to GC OS, we divided into groups based on GC patients TNM stage with information from the TCGA database. Firstly, 131 differentially expressed lncRNAs were subjected to univariate Cox proportional hazards regression, with a significance level at 0.05. A total of 23 OS-related lncRNAs were identified. Meanwhile, multivariate Cox hazards regression analysis showed that LINC01018, LOC553137, MIR4435-2HG, and TTTY14 all had a significant prognostic value for GC survival. Then, we set a risk score by combining these four lncRNAs and found that this four-lncRNA signature could independently predict OS in GC patients. The advantage of this study is a combination of clinical features and TCGA data to assess the survival of GC patients by setting a lncRNA-related risk score.

The relationship between differentially expressed lncRNAs and the survival of GC has been studied in small samples via different approaches. Li et al. [16] analyzed the prognostic value of one lncRNA via qRT-PCR array in 84 GC patients and found that higher level of BANCR could predict a poor prognosis for GC patients. Similarly, Fu et al. [25] studied lncRNA-NEAT1 in 140 freshly frozen GC samples and 20 paired adjacent normal gastric tissue samples via qRT-PCR. In addition, Fan et al. [26] has done data mining in GEO database and achieved four studies: GSE63089, GSE50710, GSE38749, and GSE27342, from which they found that AK001094, AK024171, AK093735, NR003573, and BC003519, these five lncRNAs could be considered as an independent risk factor for GC patients.

Although TCGA database has been used to analyze the lncRNA-related signature for GC prognosis [27], compared with previous studies, the advantage of this study was the combination of clinical features and TCGA data and assessed the survival of GC patients by constructing a risk score that associated with lncRNAs. Based on this, the four novel lncRNAs (LINC01018, LOC553137, MIR4435-2HG, and TTTY14) have the reason to be a new risk factor. Besides, the risk score constructed from these four lncRNAs could be served as a prognostic indicator for GC patients.

However, there is no study as of yet investigated the function of those above four lncRNAs. Here, we identified the genes that strongly correlated with the four lncRNAs expression (Pearson |R| > 0.5) in TCGA database. 434 genes were identified co-expressed with the four lncRNAs. The relevant genes were mainly enriched in synaptic transmission, transmembrane transport, Neuroactive ligand-receptor interaction and Glutamatergic synapse. After the PPI network construction, 106 co-expressed genes revealed as hub genes in the regulation of the four lncRNAs in GC.

The findings of this study may have substantial clinical significance; however, some limitations should be taken into consideration. First, we identified the target lncRNAs by using tumor stage of GC, but tumor metastasis was not included. Second, the data extracted from TCGA were based on the RNA-Seq technique; other experimental methods are required to verify the results. Third, the role of LINC01018, LOC553137, MIR4435-2HG, and TTTY14 in GC are still unknown; *in vivo* and *in vitro* experiments are expected to answer this question.

In conclusion, by analyzing the GC lncRNA expression profiles in a large-scale database from TCGA, we identified a four-lncRNA signature, which could act as an indicator for GC patient outcome and could be a potential independent biomarker for prognosis prediction of GC. Future functional investigations are required to explore the mechanisms underlying the roles of these lncRNAs in GC.

## MATERIALS AND METHODS

### TCGA database

The GC data (Level 3 RNA sequencing) of 443 individuals with clinical information were extracted from TCGA database on April 10, 2017, including data from 408 GC tissues and 35 adjacent normal gastric tissues. The exclusion criteria were listed as follows: (i) histologic diagnosis ruled out GC; (ii) another malignancy besides GC. Then, 379 GC patients were included in this study. As the data was downloaded from the public database, ethical approval was not applicable in this case. Data processing procedures met the policies of TCGA data access and human subject protection (http://cancergenome.nih.gov/publications/publicationguidelines). Of these 379 GC patients, there were 54 GC patients with tumor stage I, 120 GC patients with tumor stage II, 166 GC patients with tumor stage III and 39 GC patients with tumor stage IV.

### Identification of dysregulated lncRNAs in GC

**Figure 11:**
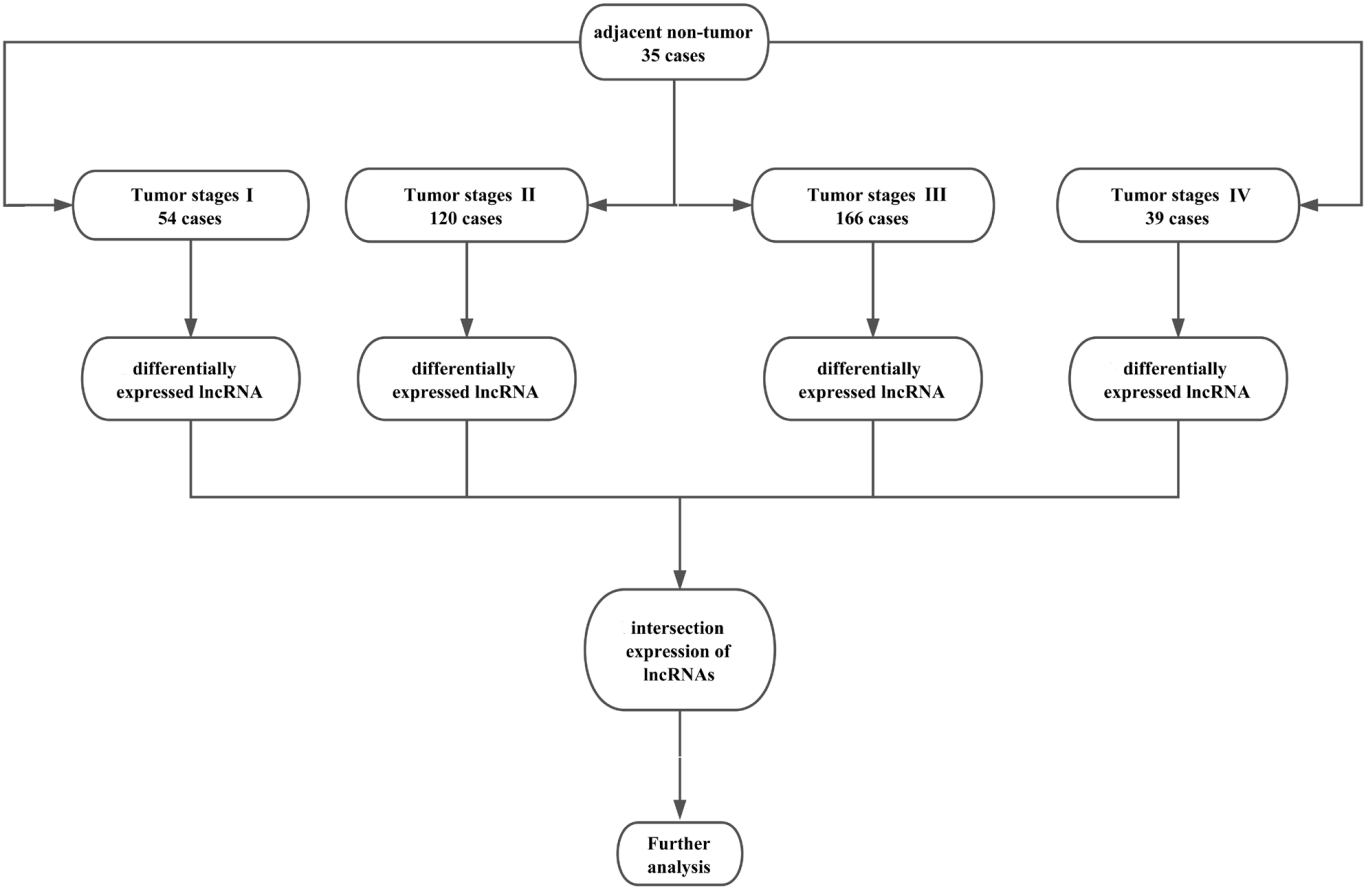
Flow chart of bioinformatics analysis

Here, only lncRNAs with a description from NCBI or Ensemble were selected for further study. Finally, we obtained the expression profiles of 1801 lncRNAs. The raw data of lncRNA sequencing were post-processed and normalized by TCGA RNASeqv2 system. No further normalizations were applied in the expression profile data in level 3, due to TCGA already normalized these data. To detect the differential expression of lncRNAs, samples were divided into GC tumor tissues vs. adjacent non-tumor gastric tissues, tumor stage I, stage II, stage III, and stage IV. For further analysis, the intersection of lncRNA was selected. The flow chart for bioinformatics analysis was presented in Figure 11.

### Construction of the prognostic signature

The GC-specific lncRNAs were selected, and the expression level of each lncRNA was log2 transformed for further analysis. The univariate Cox proportional hazards regression model was used to analyze the GC-specific lncRNAs associated with OS. The multivariate Cox regression model was further performed to evaluate the prognostic value of these OS-related lncRNAs. The semi-supervised method that combines the gene expression profile with clinical information was used to conduct univariate Cox regression analyses [28, 29]. In each subgroup stratified by tumor TNM system, the OS-related lncRNAs were identified by the multivariate Cox regression model.

The prognostic risk score for predicting OS was calculated: Risk score = exp_lncRNA1_*β_lncRNA1_ + exp_lncRNA2_*β_lncRNA2_ + …exp_lncRNAn_*β_lncRNAn_ (exp: expression level; β: the regression coefficient derived from the multivariate Cox regression model) [30]. The median risk score was used as the cutoff point, and GC patients were divided into high- and low- groups [31]. Further univariate and multivariate Cox proportional hazards regression analyses were conducted to investigate the effects of various clinical features and the risk score of OS for GC patients. The hazard ratio (HR) and 95% confidence interval (CI) were assessed. The defining point set up by 5-year time-dependent receiver operating characteristic (ROC) curve analysis, was used to evaluate the predictive value of the risk score for time-dependent outcomes [32]. Via IBM SPSS Statistics 21 (SPSS Inc., Chicago, IL, USA), Kaplan-Meier survival curves and the log-rank test were used to assess the equality of survival distributions in different groups. The ROC was used to assess GC-specific lncRNAs for the sensitivity and specificity of GC detection.

### Integrative prediction analysis of lncRNA function

The four lncRNAs expression was heterogeneous across different grade GC. To investigate the biological feature of GC with different four lncRNAs expression, we asked the genes that strongly correlated with these four lncRNAs expression (Pearson |R| > 0.5) in TCGA database [33]. The Kyoto Encyclopedia of Genes and Genomes (KEGG) and Gene Ontology (GO) enrichment analyses of co-expressed mRNAs of these lncRNAs were performed using the Database for Annotation, Visualization, and Integrated Discovery (DAVID) (https://david.ncifcrf.gov/). The enriched results were restricted to KEGG pathway and GO biological process. The adjusted P-value < 0.05 was considered to be significant. Then, the co-expressed genes were performed to construct the protein-protein interaction (PPI) network via STRING (Version 10.5) (https://string-db.org/).

## SUPPLEMENTARY MATERIALS TABLES







## Abbreviations

lncRNAs: long non-coding RNAs
GC: gastric cancer
TCGA: The Cancer Genome Atlas
OS: Overall Survival
STDEV: standard deviation
CR: complete remission
PR: partial remission
SD: stable disease
PD: progressive disease
AUC: area under ROC curve
HR: the hazard ratio
CI: confidence interval
ROC: receiver operating characteristic
KEGG: the Kyoto Encyclopedia of Genes and Genomes
GO: Gene Ontology;
DAVID: the Database for Annotation, Visualization, and Integrated Discovery
PPI: the protein-protein interaction
