# Supplementary material for: Comprehensive analysis of a novel four-lncRNA signature as a prognostic biomarker for human gastric cancer
Source: Oncotarget. 2017 Aug 24;8(43):75007–24. doi: 10.18632/oncotarget.20496 (PMC5650396; doi:10.18632/oncotarget.20496)
Supplement: Supplementary file 1 [file oncotarget-08-75007-s001.pdf]

Comprehensive analysis of a novel four-lncRNA signature as a prognostic biomarker for human gastric cancer

SUPPLEMENTARY MATERIALS

Supplementary Table 1: The co-expressed mRNAs of the four lncRNAs (LINC01018, LOC553137, MIR4435-2HG, and TTTY14)

See Supplementary File 1

Supplementary Table 2: KEGG Pathway and GO term of co-expressed mRNAs in GC

See Supplementary File 2
