# Supplementary material for: Comprehensive analysis of a novel four-lncRNA signature as a prognostic biomarker for human gastric cancer
Source: Oncotarget. 2017 Aug 24;8(43):75007–24. doi: 10.18632/oncotarget.20496 (PMC5650396; doi:10.18632/oncotarget.20496)
Supplement: Supplementary file 2 [file oncotarget-08-75007-s002.docx]

**Supplementary Table 1: The co-expressed mRNAs of the four lncRNAs (LINC01018, LOC553137, MIR4435-2HG, and TTTY14).**

| lncRNA | Co-expressed mRNA | \|Pearson\| |
| --- | --- | --- |
| \| TTTY14 \| \| --- \| \| TTTY14 \| \| TTTY14 \| \| TTTY14 \| \| TTTY14 \| \| TTTY14 \| \| TTTY14 \| \| TTTY14 \| \| TTTY14 \| \| TTTY14 \| \| TTTY14 \| \| TTTY14 \| \| TTTY14 \| \| TTTY14 \| \| TTTY14 \| \| TTTY14 \| \| TTTY14 \| \| TTTY14 \| \| TTTY14 \| \| TTTY14 \| \| TTTY14 \| \| TTTY14 \| \| TTTY14 \| \| TTTY14 \| \| TTTY14 \| \| TTTY14 \| \| TTTY14 \| \| TTTY14 \| \| TTTY14 \| \| TTTY14 \| \| TTTY14 \| \| TTTY14 \| \| TTTY14 \| \| TTTY14 \| \| TTTY14 \| \| TTTY14 \| \| TTTY14 \| \| TTTY14 \| \| TTTY14 \| \| TTTY14 \| \| TTTY14 \| \| TTTY14 \| \| TTTY14 \| \| TTTY14 \| \| TTTY14 \| \| TTTY14 \| \| TTTY14 \| \| TTTY14 \| \| TTTY14 \| \| TTTY14 \| \| TTTY14 \| \| TTTY14 \| \| TTTY14 \| \| TTTY14 \| \| TTTY14 \| \| TTTY14 \| \| TTTY14 \| \| TTTY14 \| \| TTTY14 \| \| TTTY14 \| \| TTTY14 \| \| TTTY14 \| \| TTTY14 \| \| TTTY14 \| \| TTTY14 \| \| TTTY14 \| \| TTTY14 \| \| TTTY14 \| \| TTTY14 \| \| TTTY14 \| \| TTTY14 \| \| TTTY14 \| \| TTTY14 \| \| LINC01018 \| \| LINC01018 \| \| LINC01018 \| \| LINC01018 \| \| LINC01018 \| \| LINC01018 \| \| LINC01018 \| \| LINC01018 \| \| LINC01018 \| \| LINC01018 \| \| LINC01018 \| \| LINC01018 \| \| LINC01018 \| \| LINC01018 \| \| LINC01018 \| \| LINC01018 \| \| LINC01018 \| \| LINC01018 \| \| LINC01018 \| \| LINC01018 \| \| LINC01018 \| \| LINC01018 \| \| LINC01018 \| \| LINC01018 \| \| LINC01018 \| \| LINC01018 \| \| LINC01018 \| \| LINC01018 \| \| LINC01018 \| \| LINC01018 \| \| LINC01018 \| \| LINC01018 \| \| LINC01018 \| \| LINC01018 \| \| LINC01018 \| \| LINC01018 \| \| LINC01018 \| \| LINC01018 \| \| LINC01018 \| \| LINC01018 \| \| LINC01018 \| \| LINC01018 \| \| LINC01018 \| \| LINC01018 \| \| LINC01018 \| \| LINC01018 \| \| LINC01018 \| \| LINC01018 \| \| LINC01018 \| \| LINC01018 \| \| LINC01018 \| \| LINC01018 \| \| LINC01018 \| \| LINC01018 \| \| LINC01018 \| \| LINC01018 \| \| LINC01018 \| \| LINC01018 \| \| LINC01018 \| \| LINC01018 \| \| LINC01018 \| \| LINC01018 \| \| LINC01018 \| \| LINC01018 \| \| LINC01018 \| \| LINC01018 \| \| LINC01018 \| \| LINC01018 \| \| LINC01018 \| \| LINC01018 \| \| LINC01018 \| \| LINC01018 \| \| LINC01018 \| \| LINC01018 \| \| LINC01018 \| \| LINC01018 \| \| LINC01018 \| \| LINC01018 \| \| LINC01018 \| \| LINC01018 \| \| LINC01018 \| \| LINC01018 \| \| LINC01018 \| \| LINC01018 \| \| LINC01018 \| \| LINC01018 \| \| LINC01018 \| \| LINC01018 \| \| LINC01018 \| \| LINC01018 \| \| LINC01018 \| \| LINC01018 \| \| LINC01018 \| \| LINC01018 \| \| LINC01018 \| \| LINC01018 \| \| LINC01018 \| \| LINC01018 \| \| LINC01018 \| \| LINC01018 \| \| LINC01018 \| \| LINC01018 \| \| LINC01018 \| \| LINC01018 \| \| LINC01018 \| \| LINC01018 \| \| LINC01018 \| \| LINC01018 \| \| LINC01018 \| \| LINC01018 \| \| LINC01018 \| \| LINC01018 \| \| LINC01018 \| \| LINC01018 \| \| LINC01018 \| \| LINC01018 \| \| LINC01018 \| \| LINC01018 \| \| LINC01018 \| \| LINC01018 \| \| LINC01018 \| \| LINC01018 \| \| LINC01018 \| \| LINC01018 \| \| LINC01018 \| \| LINC01018 \| \| LINC01018 \| \| LINC01018 \| \| LINC01018 \| \| LINC01018 \| \| LINC01018 \| \| LINC01018 \| \| LINC01018 \| \| LINC01018 \| \| LINC01018 \| \| LINC01018 \| \| LINC01018 \| \| LINC01018 \| \| LINC01018 \| \| LINC01018 \| \| LINC01018 \| \| LINC01018 \| \| LINC01018 \| \| LINC01018 \| \| LINC01018 \| \| LINC01018 \| \| LINC01018 \| \| LINC01018 \| \| LINC01018 \| \| LINC01018 \| \| LINC01018 \| \| LINC01018 \| \| LINC01018 \| \| LINC01018 \| \| LINC01018 \| \| LINC01018 \| \| LINC01018 \| \| LINC01018 \| \| LINC01018 \| \| LINC01018 \| \| LINC01018 \| \| LINC01018 \| \| LINC01018 \| \| LINC01018 \| \| LINC01018 \| \| LINC01018 \| \| LINC01018 \| \| LINC01018 \| \| LINC01018 \| \| LINC01018 \| \| LINC01018 \| \| LINC01018 \| \| LINC01018 \| \| LINC01018 \| \| LINC01018 \| \| LINC01018 \| \| LINC01018 \| \| LINC01018 \| \| LINC01018 \| \| LINC01018 \| \| LINC01018 \| \| LINC01018 \| \| LINC01018 \| \| LINC01018 \| \| LINC01018 \| \| LINC01018 \| \| LINC01018 \| \| LINC01018 \| \| LINC01018 \| \| LINC01018 \| \| LINC01018 \| \| LINC01018 \| \| LINC01018 \| \| LINC01018 \| \| LINC01018 \| \| LINC01018 \| \| LINC01018 \| \| LINC01018 \| \| LINC01018 \| \| LINC01018 \| \| LINC01018 \| \| LINC01018 \| \| LINC01018 \| \| LINC01018 \| \| LINC01018 \| \| MIR4435-2HG \| \| MIR4435-2HG \| \| MIR4435-2HG \| \| MIR4435-2HG \| \| MIR4435-2HG \| \| MIR4435-2HG \| \| MIR4435-2HG \| \| MIR4435-2HG \| \| MIR4435-2HG \| \| MIR4435-2HG \| \| MIR4435-2HG \| \| MIR4435-2HG \| \| MIR4435-2HG \| \| MIR4435-2HG \| \| MIR4435-2HG \| \| MIR4435-2HG \| \| MIR4435-2HG \| \| MIR4435-2HG \| \| MIR4435-2HG \| \| MIR4435-2HG \| \| LOC553137 \| \| LOC553137 \| \| LOC553137 \| \| LOC553137 \| \| LOC553137 \| \| LOC553137 \| \| LOC553137 \| \| LOC553137 \| \| LOC553137 \| \| LOC553137 \| \| LOC553137 \| \| LOC553137 \| \| LOC553137 \| \| LOC553137 \| \| LOC553137 \| \| LOC553137 \| \| LOC553137 \| \| LOC553137 \| \| LOC553137 \| \| LOC553137 \| \| LOC553137 \| \| LOC553137 \| \| LOC553137 \| \| LOC553137 \| \| LOC553137 \| \| LOC553137 \| \| LOC553137 \| \| LOC553137 \| \| LOC553137 \| \| LOC553137 \| \| LOC553137 \| \| LOC553137 \| \| LOC553137 \| \| LOC553137 \| \| LOC553137 \| \| LOC553137 \| \| LOC553137 \| \| LOC553137 \| \| LOC553137 \| \| LOC553137 \| \| LOC553137 \| \| LOC553137 \| \| LOC553137 \| \| LOC553137 \| \| LOC553137 \| \| LOC553137 \| \| LOC553137 \| \| LOC553137 \| \| LOC553137 \| \| LOC553137 \| \| LOC553137 \| \| LOC553137 \| \| LOC553137 \| \| LOC553137 \| \| LOC553137 \| \| LOC553137 \| \| LOC553137 \| \| LOC553137 \| \| LOC553137 \| \| LOC553137 \| \| LOC553137 \| \| LOC553137 \| \| LOC553137 \| \| LOC553137 \| \| LOC553137 \| \| LOC553137 \| \| LOC553137 \| \| LOC553137 \| \| LOC553137 \| \| LOC553137 \| \| LOC553137 \| \| LOC553137 \| \| LOC553137 \| \| LOC553137 \| \| LOC553137 \| \| LOC553137 \| \| LOC553137 \| \| LOC553137 \| \| LOC553137 \| \| LOC553137 \| \| LOC553137 \| \| LOC553137 \| \| LOC553137 \| \| LOC553137 \| \| LOC553137 \| \| LOC553137 \| \| LOC553137 \| \| LOC553137 \| \| LOC553137 \| \| LOC553137 \| \| LOC553137 \| \| LOC553137 \| \| LOC553137 \| \| LOC553137 \| \| LOC553137 \| \| LOC553137 \| \| LOC553137 \| \| LOC553137 \| \| LOC553137 \| \| LOC553137 \| \| LOC553137 \| \| LOC553137 \| \| LOC553137 \| \| LOC553137 \| \| LOC553137 \| \| LOC553137 \| \| LOC553137 \| \| LOC553137 \| \| LOC553137 \| \| LOC553137 \| \| LOC553137 \| \| LOC553137 \| \| LOC553137 \| \| LOC553137 \| \| LOC553137 \| \| LOC553137 \| \| LOC553137 \| \| LOC553137 \| \| LOC553137 \| \| LOC553137 \| \| LOC553137 \| \| LOC553137 \| \| LOC553137 \| \| LOC553137 \| \| LOC553137 \| \| LOC553137 \| \| LOC553137 \| \| LOC553137 \| \| LOC553137 \| \| LOC553137 \| \| LOC553137 \| \| LOC553137 \| \| LOC553137 \| \| LOC553137 \| \| LOC553137 \| \| LOC553137 \| | ATP4B  COX7A2  COX7B  CRYGD  CYB5A  DAZ1  GAST  BLOC1S1  GSTA3  HBM  HBB  SCGB2A1  NDUFA2  NDUFA4  NDUFB7  NDUFS4  PDE4C  PFDN5  PXMP2  RPL41  RPS4Y1  RPS10  S100P  TFF1  TFF2  CLEC3B  PLA2G10  CRADD  SNURF  EIF1AY  TMSB4Y  MRPL33  KCNE2  SERF2  LYVE1  UQCR11  PRR4  VSIG2  HIGD1A  UQCRQ  UQCR10  PLLP  SMIM11A  HMGCLL1  GKN1  DAZ3  MMP27  C19orf33  FUNDC2  PRADC1  CYSTM1  COX14  TMEM261  SMDT1  C11orf52  C12orf57  MRPL54  TMEM42  C16orf89  C22orf42  COPS9  LCN6  GKN2  PLAC9  TMEM211  TRIM74  REP15  SMIM20  PTRHD1  DEFB132  SMIM5  OST4  SMIM6  ASIC2  ADCY1  ADCY2  ADCYAP1  AOX1  AIRE  ASCL1  ATP1A3  KIF1A  CACNA1A  CALCA  CGA  CHRNB2  CRMP1  DBH  DNAH5  DRP2  ELAVL4  GHRH  GLRB  GNG4  GNRH2  GPR6  GRIA2  GRIK2  GRIN2C  GRM4  GRM5  GRP  GRPR  GUCY2C  HAL  HCRT  KCNJ6  KIF5C  IPO5  MAN1A1  MAP2  MAPT  NCAM1  OPCML  OXCT1  PCSK2  PLXNA2  POU3F1  POU4F1  POU4F2  RFX4  RFXAP  SCN3A  SH3GL2  SLC8A3  SNAP25  SNCB  SPTB  SYT4  TACR1  TNR  BSND  ALDH5A1  DPF1  IRS2  ARHGEF7  CPNE3  BSN  WASF1  MTMR7  CBFA2T2  NEURL1  STXBP5L  RIMS2  ST18  MTSS1  DNAJC6  KIAA0196  SV2B  FRY  KCNMB2  SCGB1D2  DLL3  RUNDC3A  SLC38A3  ANKRD6  BTBD3  MAST1  UNC13A  PHF8  MTUS2  ATRNL1  ERC2  RGS17  NBEA  FAM184B  RPS6KA6  CECR6  SCG3  DDX25  DNMT3L  KLK14  KLK12  ASAP1  RMDN1  RXFP3  HMP19  ATP8A2  SNTG2  RBFOX1  RAB39A  ELOVL2  SLC38A4  SCN3B  PRMT8  RPRM  MMP26  DPYSL5  SLC17A6  CAMK1D  TMEM63C  LRFN2  TXNDC16  UNC79  PITPNM2  FAM234B  NGB  NEUROD4  DLGAP3  NAPB  LHX5  NDST4  DUSP26  TRPM8  CERS4  PGBD5  ZNF322  ZMAT4  TTLL7  TREML2  BAALC  NRSN2  TRIM46  ASXL3  OR51G2  FSD1L  SLITRK6  SRRM4  TRIM55  INSM2  SLC22A16  PSKH2  KCNH7  SEC11C  DNER  PHF21B  KCTD12  RAB39B  CLNK  DACH2  SPACA7  LOXHD1  SGSM1  UBE2QL1  RIPPLY2  SPIN4  NRSN1  SYT9  OR51E1  TSPAN19  BEST3  CBLN2  PNPLA5  PLEKHG4B  CNKSR3  FAM84B  SAXO1  ZDHHC15  EML5  CABP7  KCNG3  VWDE  MTURN  SERPINA11  HS6ST3  DPY19L2  KSR2  ZDHHC22  TMEM145  C20orf197  UNC80  NSMCE2  XKR7  ZACN  SOGA3  SLC6A17  GLYATL3  SKOR1  CTXN2  FOXI2  SERTM1  OR14I1  VWC2L  TRIM67  PIWIL3  TMEM151B  FOXB2  PIRT  CNN2  CSF2  IL15RA  KRT7  S100A11  FOSL1  TMSB10  IFITM3  SSSCA1  BATF  DRAP1  CCDC85B  BRMS1  PPP1R14B  MYEOV  RHOD  TNFRSF12A  FXYD5  CATSPER1  LCE3B  ACTN3  ADRA1B  ASTN1  ATP2B2  CALCR  CDH18  CHGA  CHGB  CPE  EDA  ELAVL2  FGL1  GAD2  GHSR  GRID2  HPCA  INSM1  LALBA  LMX1A  MMP16  NEUROD1  NEUROD2  NKX2-2  NTN3  OPRK1  PAK3  PAX4  PCM1  SERPINI1  PYY  PTPRN  SLC7A2  SLC18A1  SLC18A2  ELOVL4  STXBP1  TPH1  CRISP2  TSHB  USH2A  VGF  TFPI2  TUSC3  BSN  KCNB2  ABCG1  SLC17A2  LBX1  C6orf10  PTPRT  PPM1E  EPB41L3  SMC5  ABCB10  RIMBP2  LRRTM2  CFAP61  OR2M4  SERGEF  CACNG5  DKKL1  CNOT7  ABHD17B  LRP1B  PRKAG3  FEV  TRIM39  SLC17A7  HRASLS  MTUS1  KCTD16  MARCH4  NYAP2  DPP10  BEND3  CCDC181  PAPPA2  GFRA4  MTMR9  JADE1  CXXC4  FAM167A  CCNB3  FATE1  VWA5B2  TGIF2LX  TGIF2LY  L3MBTL4  KCNG4  PTH2  FAM122A  MED12L  WDR17  C10orf71  KIF19  SEZ6  OR2M3  OR2T4  ADAD1  TAAR1  C6orf141  VPS37A  SGCZ  C9orf57  C9orf85  C9orf135  BHLHA15  HTR3C  PLD5  TCTE1  FAM181B  RFX6  TMEM196  IGSF10  GPRIN3  C7orf71  TRIM42  TGM6  GPR148  PLCXD3  LRRTM3  C22orf34  GPR142  PTAR1  AMIGO3  RPRML  GLYATL3  TMEM8C  PAGE2B  SLC25A53  APOBEC4  LHX8  FAM47C  TMEM200C  CFC1B  KHDC1L | 0.511480889  0.582631756  0.537526426  0.679384471  0.590612379  0.659484697  0.75220871  0.557884928  0.777735198  0.627476749  0.571978417  0.702000642  0.542191966  0.610960492  0.525672853  0.552923525  0.530403542  0.538783146  0.557014868  0.60702301  0.636565295  0.525799512  0.552472625  0.652817627  0.684207507  0.652517245  0.541085779  0.52557772  0.506879592  0.541493921  0.510730287  0.532473181  0.728336276  0.508007861  0.509154147  0.63163742  0.685428618  0.545799431  0.543620421  0.55123514  0.552470299  0.532350707  0.593681322  0.526589813  0.693495725  0.504224277  0.558777845  0.513361903  0.552381254  0.55673874  0.661333958  0.542621567  0.519761992  0.577783784  0.564589011  0.59810758  0.576852454  0.535397554  0.661075379  0.536009274  0.547334391  0.637951048  0.70073661  0.552402145  0.501435976  0.668431983  0.582476727  0.516624217  0.569634305  0.687882476  0.526097704  0.540783304  0.650962222  0.511440108  0.763549104  0.65341314  0.591045181  0.725586621  0.614978803  0.775760014  0.810361848  0.804841907  0.710997128  0.774414357  0.730781356  0.561858815  0.597785177  0.578980315  0.738563634  0.720635426  0.751155513  0.741554358  0.536449125  0.536371127  0.754699866  0.676259655  0.514112  0.510238756  0.727201373  0.608136127  0.568118944  0.671718732  0.523532732  0.503177421  0.7360014  0.544140916  0.574488909  0.838948793  0.527699292  0.664424297  0.541896591  0.576372612  0.535535759  0.743651422  0.655742504  0.745777579  0.569282203  0.717405434  0.75164237  0.635213554  0.740725955  0.550131849  0.787892352  0.817946136  0.744948817  0.627028272  0.766259034  0.648768586  0.720789359  0.649948262  0.582934367  0.759502976  0.610595697  0.704996053  0.54530071  0.56596013  0.572995001  0.537160563  0.540683033  0.621785851  0.522304386  0.532842382  0.768233402  0.825758389  0.838854546  0.547300867  0.632487373  0.597731756  0.515726679  0.523037698  0.756885057  0.588146316  0.719073255  0.533092775  0.575827276  0.569289332  0.774573953  0.677752633  0.706087392  0.644332245  0.839558981  0.567977961  0.543396791  0.73747689  0.629383554  0.517503804  0.601234943  0.77709221  0.60097063  0.719123981  0.603144536  0.687643069  0.701036907  0.659019969  0.665769707  0.655432471  0.63842875  0.732368721  0.524367633  0.750534897  0.575862835  0.746420739  0.578369231  0.565020059  0.559412377  0.589603772  0.567797971  0.76897597  0.745754117  0.700974713  0.767686968  0.586531683  0.67979107  0.796576257  0.542434732  0.605217567  0.776368686  0.535086709  0.531434594  0.542327934  0.618350646  0.751060208  0.579471793  0.667940857  0.634720329  0.511215355  0.651207447  0.632129597  0.53201503  0.546369806  0.732713243  0.523753452  0.508036239  0.705811662  0.68738238  0.565642052  0.524085536  0.790238735  0.516992292  0.739283729  0.691636679  0.766460098  0.691980932  0.573366712  0.565988964  0.814699272  0.572997315  0.506659921  0.728361411  0.721958959  0.762430025  0.689588328  0.78867275  0.835939068  0.603880857  0.64239556  0.785816551  0.568266683  0.716097741  0.709038746  0.573270019  0.502023717  0.646931381  0.638263001  0.753317284  0.688278977  0.64136316  0.614164055  0.659936182  0.748011347  0.501185638  0.662448546  0.534970922  0.50910573  0.504215581  0.50832774  0.681942961  0.629135018  0.71467901  0.682042324  0.664124994  0.555721571  0.511799141  0.763467535  0.669593539  0.761039449  0.523078501  0.681708941  0.561486623  0.525366657  0.518391364  0.745866081  0.517674207  0.549297387  0.520300334  0.577469029  0.742446794  0.640150415  0.573249579  0.506183035  0.532942852  0.505187286  0.544399864  0.516962231  0.565990485  0.500324683  0.518718581  0.542700605  0.55841669  0.51704447  0.528662411  0.547840495  0.557560415  0.543193985  0.532764989  0.585403936  0.50242851  0.503420163  0.877640579  0.923577351  0.614563361  0.670826311  0.914567901  0.840696691  0.834680822  0.825736421  0.726168562  0.500876602  0.740601819  0.794506286  0.845221637  0.809114704  0.592074814  0.916766108  0.75814935  0.920545956  0.922879667  0.50387125  0.55471584  0.92257199  0.843858337  0.50400223  0.655631176  0.818354403  0.643206039  0.606365836  0.800516965  0.686121291  0.65499877  0.916192352  0.852450539  0.883464864  0.742338762  0.749415788  0.923727412  0.674121706  0.570921725  0.507306514  0.577658761  0.862328371  0.777760364  0.576270306  0.679209792  0.57882218  0.607563729  0.64528299  0.598987742  0.697316161  0.629109487  0.675968584  0.565494496  0.558472859  0.590770705  0.654328332  0.741431064  0.891914035  0.579439217  0.787711671  0.718790861  0.582749305  0.545266522  0.742330183  0.887537849  0.87784596  0.549487986  0.784199368  0.539290833  0.703720553  0.795410657  0.607712818  0.90000474  0.510030859  0.890299567  0.628779167  0.921532531  0.725777249  0.567686007  0.532999862  0.526195526  0.911138147  0.651186988  0.812798839  0.905590769  0.888130773  0.725909258  0.773569085  0.868834114  0.724322018  0.585900349  0.564352153  0.590486491  0.696871442  0.835924852  0.605933802  0.8583419  0.919071469  0.637383364  0.559070373  0.527538033  0.678492782  0.709776588  0.903170869  0.595268158  0.570892471  0.566303453  0.902206522  0.699898334  0.727729732  0.812433549  0.746697064  0.7877218  0.505459051  0.593343896  0.893852076  0.69842426  0.764010778  0.821874958  0.671345957  0.92341927  0.608367407  0.743414031  0.616915881  0.801213492  0.580653804  0.618461312  0.560828737  0.821563836  0.63151781  0.504373494  0.572882288  0.565178741  0.759431728  0.859273274  0.651678468 |
