# Supplementary material for: Comprehensive analysis of a novel four-lncRNA signature as a prognostic biomarker for human gastric cancer
Source: Oncotarget. 2017 Aug 24;8(43):75007–24. doi: 10.18632/oncotarget.20496 (PMC5650396; doi:10.18632/oncotarget.20496)
Supplement: Supplementary file 3 [file oncotarget-08-75007-s003.docx]

**Supplementery Table 2: KEGG Pathway and GO term of co-expressed mRNAs in GC**

| **Category** | **ID** | **Term** | **No. of Genes** | **enrichment** | **-lgP** | **FDR** |
| --- | --- | --- | --- | --- | --- | --- |
| **GO** | GO:0007268 | synaptic transmission | 30 | 10.824 | 20.49332 | 0.000 |
|  | GO:0055085 | transmembrane transport | 23 | 5.923 | 10.19441 | 0.000 |
|  | GO:0007269 | neurotransmitter secretion | 8 | 21.314 | 7.802724 | 0.000 |
|  | GO:0022904 | respiratory electron transport chain | 9 | 14.499 | 7.258719 | 0.000 |
|  | GO:0050808 | synapse organization | 6 | 34.635 | 7.181223 | 0.000 |
|  | GO:0060079 | regulation of excitatory postsynaptic membrane potential | 6 | 31.971 | 6.953398 | 0.000 |
|  | GO:0042593 | glucose homeostasis | 8 | 14.778 | 6.514988 | 0.000 |
|  | GO:0006814 | sodium ion transport | 8 | 14.583 | 6.469414 | 0.000 |
|  | GO:0007417 | central nervous system development | 9 | 11.233 | 6.286758 | 0.000 |
|  | GO:0034220 | ion transmembrane transport | 9 | 10.567 | 6.058226 | 0.000 |
|  | GO:0035249 | synaptic transmission, glutamatergic | 5 | 34.635 | 5.964221 | 0.000 |
|  | GO:0007186 | G-protein coupled receptor signaling pathway | 13 | 5.754 | 5.626255 | 0.000 |
|  | GO:0007626 | locomotory behavior | 7 | 14.262 | 5.588255 | 0.000 |
|  | GO:0007411 | axon guidance | 13 | 5.682 | 5.564413 | 0.000 |
|  | GO:0007165 | signal transduction | 24 | 3.228 | 5.552916 | 0.000 |
|  | GO:0048665 | neuron fate specification | 4 | 50.379 | 5.469184 | 0.000 |
|  | GO:0044281 | small molecule metabolic process | 28 | 2.846 | 5.396338 | 0.000 |
|  | GO:0007399 | nervous system development | 12 | 5.875 | 5.287227 | 0.000 |
|  | GO:0050796 | regulation of insulin secretion | 7 | 12.595 | 5.217922 | 0.000 |
|  | GO:0007275 | multicellular organismal development | 15 | 4.258 | 4.854469 | 0.001 |
|  | GO:0006811 | ion transport | 9 | 7.557 | 4.837775 | 0.001 |
|  | GO:0030279 | negative regulation of ossification | 4 | 34.635 | 4.74006 | 0.001 |
|  | GO:0008306 | associative learning | 4 | 30.787 | 4.51938 | 0.001 |
|  | GO:0031018 | endocrine pancreas development | 5 | 17.762 | 4.443446 | 0.002 |
|  | GO:0007204 | elevation of cytosolic calcium ion concentration | 7 | 9.325 | 4.34433 | 0.002 |
|  | GO:0007189 | adenylate cyclase-activating G-protein coupled receptor signaling pathway | 5 | 16.895 | 4.334124 | 0.002 |
|  | GO:0007267 | cell-cell signaling | 10 | 5.725 | 4.30726 | 0.002 |
|  | GO:0016188 | synaptic vesicle maturation | 3 | 59.375 | 4.290973 | 0.002 |
|  | GO:0006351 | transcription, DNA-dependent | 31 | 2.351 | 4.245128 | 0.002 |
|  | GO:0007409 | axonogenesis | 6 | 11.233 | 4.174732 | 0.002 |
|  | GO:0030154 | cell differentiation | 12 | 4.352 | 3.973852 | 0.004 |
|  | GO:0045597 | positive regulation of cell differentiation | 4 | 22.167 | 3.920386 | 0.004 |
|  | GO:0003326 | pancreatic A cell fate commitment | 2 | 138.542 | 3.6821 | 0.006 |
|  | GO:0003329 | pancreatic PP cell fate commitment | 2 | 138.542 | 3.6821 | 0.006 |
|  | GO:0030070 | insulin processing | 2 | 138.542 | 3.6821 | 0.006 |
|  | GO:0034184 | positive regulation of maintenance of mitotic sister chromatid cohesion | 2 | 138.542 | 3.6821 | 0.006 |
|  | GO:0035881 | amacrine cell differentiation | 2 | 138.542 | 3.6821 | 0.006 |
|  | GO:0006810 | transport | 11 | 4.354 | 3.642915 | 0.007 |
|  | GO:0007218 | neuropeptide signaling pathway | 6 | 9.035 | 3.636767 | 0.007 |
|  | GO:0030073 | insulin secretion | 4 | 17.876 | 3.539519 | 0.008 |
|  | GO:0006112 | energy reserve metabolic process | 6 | 8.482 | 3.483405 | 0.009 |
|  | GO:0007215 | glutamate receptor signaling pathway | 3 | 31.971 | 3.39269 | 0.011 |
|  | GO:0007612 | learning | 4 | 15.833 | 3.328249 | 0.012 |
|  | GO:0045666 | positive regulation of neuron differentiation | 5 | 10.339 | 3.290775 | 0.013 |
|  | GO:0008542 | visual learning | 4 | 15.394 | 3.279571 | 0.013 |
|  | GO:0010807 | regulation of synaptic vesicle priming | 2 | 92.361 | 3.207064 | 0.015 |
|  | GO:0021530 | spinal cord oligodendrocyte cell fate specification | 2 | 92.361 | 3.207064 | 0.015 |
|  | GO:0006120 | mitochondrial electron transport, NADH to ubiquinone | 4 | 14.583 | 3.186525 | 0.015 |
|  | GO:0051591 | response to cAMP | 4 | 14.583 | 3.186525 | 0.015 |
|  | GO:0050885 | neuromuscular process controlling balance | 4 | 14.209 | 3.142 | 0.016 |
|  | GO:0007605 | sensory perception of sound | 6 | 7.044 | 3.040305 | 0.020 |
|  | GO:0019933 | cAMP-mediated signaling | 3 | 24.449 | 3.025874 | 0.020 |
|  | GO:0008277 | regulation of G-protein coupled receptor protein signaling pathway | 4 | 13.194 | 3.015608 | 0.020 |
|  | GO:0007623 | circadian rhythm | 4 | 12.888 | 2.975674 | 0.022 |
|  | GO:0014047 | glutamate secretion | 3 | 23.090 | 2.949022 | 0.023 |
|  | GO:0007420 | brain development | 7 | 5.574 | 2.930412 | 0.023 |
|  | GO:0051963 | regulation of synapse assembly | 2 | 69.271 | 2.908118 | 0.024 |
|  | GO:0001662 | behavioral fear response | 3 | 21.875 | 2.876718 | 0.025 |
|  | GO:0042755 | eating behavior | 3 | 20.781 | 2.808465 | 0.029 |
|  | GO:0045665 | negative regulation of neuron differentiation | 4 | 11.545 | 2.790267 | 0.030 |
|  | GO:0044267 | cellular protein metabolic process | 12 | 3.228 | 2.767394 | 0.031 |
|  | GO:0010976 | positive regulation of neuron projection development | 4 | 11.310 | 2.75575 | 0.031 |
|  | GO:0007616 | long-term memory | 3 | 19.792 | 2.743845 | 0.032 |
|  | GO:0006813 | potassium ion transport | 5 | 7.872 | 2.736372 | 0.032 |
|  | GO:0045842 | positive regulation of mitotic metaphase/anaphase transition | 2 | 55.417 | 2.688352 | 0.035 |
|  | GO:0051899 | membrane depolarization | 3 | 18.892 | 2.682502 | 0.035 |
|  | GO:0016044 | cellular membrane organization | 6 | 5.813 | 2.595077 | 0.042 |
|  | GO:0007586 | digestion | 4 | 10.076 | 2.563792 | 0.045 |
|  | GO:0006928 | cellular component movement | 5 | 7.141 | 2.543165 | 0.045 |
|  | GO:0001504 | neurotransmitter uptake | 2 | 46.181 | 2.514342 | 0.045 |
|  | GO:0015844 | monoamine transport | 2 | 46.181 | 2.514342 | 0.045 |
|  | GO:0016081 | synaptic vesicle docking involved in exocytosis | 2 | 46.181 | 2.514342 | 0.045 |
|  | GO:0021879 | forebrain neuron differentiation | 2 | 46.181 | 2.514342 | 0.045 |
|  | GO:0038003 | opioid receptor signaling pathway | 2 | 46.181 | 2.514342 | 0.045 |
|  | GO:0045762 | positive regulation of adenylate cyclase activity | 2 | 46.181 | 2.514342 | 0.045 |
|  | GO:0008219 | cell death | 6 | 5.469 | 2.456765 | 0.051 |
|  | GO:0042594 | response to starvation | 3 | 15.394 | 2.415516 | 0.055 |
|  | GO:0006208 | pyrimidine nucleobase catabolic process | 2 | 39.583 | 2.370295 | 0.056 |
|  | GO:0021953 | central nervous system neuron differentiation | 2 | 39.583 | 2.370295 | 0.056 |
|  | GO:0042761 | very long-chain fatty acid biosynthetic process | 2 | 39.583 | 2.370295 | 0.056 |
|  | GO:0043030 | regulation of macrophage activation | 2 | 39.583 | 2.370295 | 0.056 |
|  | GO:0048149 | behavioral response to ethanol | 2 | 39.583 | 2.370295 | 0.056 |
|  | GO:0048172 | regulation of short-term neuronal synaptic plasticity | 2 | 39.583 | 2.370295 | 0.056 |
|  | GO:0007010 | cytoskeleton organization | 5 | 6.535 | 2.369511 | 0.056 |
|  | GO:0001975 | response to amphetamine | 3 | 14.844 | 2.368619 | 0.056 |
|  | GO:0007219 | Notch signaling pathway | 5 | 6.414 | 2.333233 | 0.060 |
|  | GO:0007169 | transmembrane receptor protein tyrosine kinase signaling pathway | 4 | 8.659 | 2.315723 | 0.062 |
|  | GO:0030252 | growth hormone secretion | 2 | 34.635 | 2.247435 | 0.069 |
|  | GO:0042116 | macrophage activation | 2 | 34.635 | 2.247435 | 0.069 |
|  | GO:0042989 | sequestering of actin monomers | 2 | 34.635 | 2.247435 | 0.069 |
|  | GO:0060124 | positive regulation of growth hormone secretion | 2 | 34.635 | 2.247435 | 0.069 |
|  | GO:0030901 | midbrain development | 3 | 13.407 | 2.238193 | 0.070 |
|  | GO:0006417 | regulation of translation | 4 | 8.150 | 2.217747 | 0.072 |
|  | GO:0072659 | protein localization to plasma membrane | 3 | 12.988 | 2.197758 | 0.075 |
|  | GO:0007193 | adenylate cyclase-inhibiting G-protein coupled receptor signaling pathway | 3 | 12.595 | 2.158683 | 0.079 |
|  | GO:0030317 | sperm motility | 3 | 12.595 | 2.158683 | 0.079 |
|  | GO:0007018 | microtubule-based movement | 4 | 7.805 | 2.148434 | 0.079 |
|  | GO:0010226 | response to lithium ion | 2 | 30.787 | 2.140368 | 0.079 |
|  | GO:0015671 | oxygen transport | 2 | 30.787 | 2.140368 | 0.079 |
|  | GO:0021702 | cerebellar Purkinje cell differentiation | 2 | 30.787 | 2.140368 | 0.079 |
|  | GO:0032094 | response to food | 2 | 30.787 | 2.140368 | 0.079 |
|  | GO:0035235 | ionotropic glutamate receptor signaling pathway | 2 | 30.787 | 2.140368 | 0.079 |
|  | GO:0030819 | positive regulation of cAMP biosynthetic process | 3 | 12.224 | 2.120882 | 0.082 |
|  | GO:0008150 | biological_process | 12 | 2.690 | 2.092945 | 0.086 |
|  | GO:0071377 | cellular response to glucagon stimulus | 3 | 11.875 | 2.084279 | 0.087 |
|  | GO:0051091 | positive regulation of sequence-specific DNA binding transcription factor activity | 4 | 7.389 | 2.061005 | 0.091 |
|  | GO:0007200 | phospholipase C-activating G-protein coupled receptor signaling pathway | 3 | 11.545 | 2.048805 | 0.092 |
|  | GO:0003323 | type B pancreatic cell development | 2 | 27.708 | 2.045535 | 0.092 |
|  | GO:0006996 | organelle organization | 2 | 27.708 | 2.045535 | 0.092 |
|  | GO:0045471 | response to ethanol | 4 | 7.197 | 2.019256 | 0.097 |
|  | GO:0016042 | lipid catabolic process | 4 | 7.015 | 1.978723 | 0.105 |
|  | GO:0006171 | cAMP biosynthetic process | 2 | 25.189 | 1.96046 | 0.107 |
|  | GO:0043267 | negative regulation of potassium ion transport | 2 | 25.189 | 1.96046 | 0.107 |
|  | GO:0060384 | innervation | 2 | 25.189 | 1.96046 | 0.107 |
|  | GO:0006865 | amino acid transport | 3 | 10.657 | 1.948543 | 0.109 |
|  | GO:0001756 | somitogenesis | 3 | 10.391 | 1.916996 | 0.115 |
|  | GO:0006892 | post-Golgi vesicle-mediated transport | 3 | 10.391 | 1.916996 | 0.115 |
|  | GO:0006508 | proteolysis | 10 | 2.839 | 1.894711 | 0.119 |
|  | GO:0007158 | neuron cell-cell adhesion | 2 | 23.090 | 1.883353 | 0.119 |
|  | GO:0043568 | positive regulation of insulin-like growth factor receptor signaling pathway | 2 | 23.090 | 1.883353 | 0.119 |
|  | GO:0086005 | regulation of ventricular cardiac muscle cell action potential | 2 | 23.090 | 1.883353 | 0.119 |
|  | GO:0008284 | positive regulation of cell proliferation | 9 | 3.034 | 1.882745 | 0.119 |
|  | GO:0007613 | memory | 3 | 9.896 | 1.856431 | 0.125 |
|  | GO:0019722 | calcium-mediated signaling | 3 | 9.896 | 1.856431 | 0.125 |
|  | GO:0022900 | electron transport chain | 3 | 9.666 | 1.827331 | 0.132 |
|  | GO:0007405 | neuroblast proliferation | 2 | 21.314 | 1.812875 | 0.134 |
|  | GO:0051480 | cytosolic calcium ion homeostasis | 2 | 21.314 | 1.812875 | 0.134 |
|  | GO:0051482 | elevation of cytosolic calcium ion concentration involved in phospholipase C-activating G-protein coupled signaling pathway | 2 | 21.314 | 1.812875 | 0.134 |
|  | GO:0042391 | regulation of membrane potential | 3 | 9.446 | 1.79897 | 0.137 |
|  | GO:0042493 | response to drug | 7 | 3.539 | 1.795431 | 0.137 |
|  | GO:0019233 | sensory perception of pain | 3 | 9.236 | 1.771314 | 0.144 |
|  | GO:0010744 | positive regulation of macrophage derived foam cell differentiation | 2 | 19.792 | 1.748 | 0.148 |
|  | GO:0021527 | spinal cord association neuron differentiation | 2 | 19.792 | 1.748 | 0.148 |
|  | GO:0050768 | negative regulation of neurogenesis | 2 | 19.792 | 1.748 | 0.148 |
|  | GO:0009653 | anatomical structure morphogenesis | 4 | 5.959 | 1.724401 | 0.150 |
|  | GO:0060041 | retina development in camera-type eye | 3 | 8.843 | 1.71799 | 0.150 |
|  | GO:0035556 | intracellular signal transduction | 7 | 3.415 | 1.711589 | 0.150 |
|  | GO:0007243 | intracellular protein kinase cascade | 4 | 5.895 | 1.707954 | 0.150 |
|  | GO:0051291 | protein heterooligomerization | 3 | 8.659 | 1.692266 | 0.150 |
|  | GO:0043524 | negative regulation of neuron apoptotic process | 4 | 5.833 | 1.691709 | 0.150 |
|  | GO:0010001 | glial cell differentiation | 2 | 18.472 | 1.687922 | 0.150 |
|  | GO:0021542 | dentate gyrus development | 2 | 18.472 | 1.687922 | 0.150 |
|  | GO:0021954 | central nervous system neuron development | 2 | 18.472 | 1.687922 | 0.150 |
|  | GO:0043011 | myeloid dendritic cell differentiation | 2 | 18.472 | 1.687922 | 0.150 |
|  | GO:0043523 | regulation of neuron apoptotic process | 2 | 18.472 | 1.687922 | 0.150 |
|  | GO:0051260 | protein homooligomerization | 5 | 4.528 | 1.678833 | 0.150 |
|  | GO:0006303 | double-strand break repair via nonhomologous end joining | 2 | 17.318 | 1.632 | 0.150 |
|  | GO:0019228 | regulation of action potential in neuron | 2 | 17.318 | 1.632 | 0.150 |
|  | GO:0019229 | regulation of vasoconstriction | 2 | 17.318 | 1.632 | 0.150 |
|  | GO:0051968 | positive regulation of synaptic transmission, glutamatergic | 2 | 17.318 | 1.632 | 0.150 |
|  | GO:0001764 | neuron migration | 4 | 5.487 | 1.598263 | 0.150 |
|  | GO:0016486 | peptide hormone processing | 2 | 16.299 | 1.57971 | 0.150 |
|  | GO:0034199 | activation of protein kinase A activity | 2 | 16.299 | 1.57971 | 0.150 |
|  | GO:0035338 | long-chain fatty-acyl-CoA biosynthetic process | 2 | 16.299 | 1.57971 | 0.150 |
|  | GO:0045773 | positive regulation of axon extension | 2 | 16.299 | 1.57971 | 0.150 |
|  | GO:0048265 | response to pain | 2 | 16.299 | 1.57971 | 0.150 |
|  | GO:0050890 | cognition | 2 | 16.299 | 1.57971 | 0.150 |
|  | GO:0009611 | response to wounding | 3 | 7.842 | 1.572022 | 0.150 |
|  | GO:0001984 | vasodilation of artery involved in baroreceptor response to increased systemic arterial blood pressure | 1 | 138.542 | 1.53952 | 0.150 |
|  | GO:0001987 | vasoconstriction of artery involved in baroreceptor response to lowering of systemic arterial blood pressure | 1 | 138.542 | 1.53952 | 0.150 |
|  | GO:0002443 | leukocyte mediated immunity | 1 | 138.542 | 1.53952 | 0.150 |
|  | GO:0002878 | negative regulation of acute inflammatory response to non-antigenic stimulus | 1 | 138.542 | 1.53952 | 0.150 |
|  | GO:0003026 | regulation of systemic arterial blood pressure by aortic arch baroreceptor feedback | 1 | 138.542 | 1.53952 | 0.150 |
|  | GO:0003311 | pancreatic D cell differentiation | 1 | 138.542 | 1.53952 | 0.150 |
|  | GO:0003327 | type B pancreatic cell fate commitment | 1 | 138.542 | 1.53952 | 0.150 |
|  | GO:0003359 | noradrenergic neuron fate commitment | 1 | 138.542 | 1.53952 | 0.150 |
|  | GO:0006083 | acetate metabolic process | 1 | 138.542 | 1.53952 | 0.150 |
|  | GO:0006867 | asparagine transport | 1 | 138.542 | 1.53952 | 0.150 |
|  | GO:0007400 | neuroblast fate determination | 1 | 138.542 | 1.53952 | 0.150 |
|  | GO:0009720 | detection of hormone stimulus | 1 | 138.542 | 1.53952 | 0.150 |
|  | GO:0015817 | histidine transport | 1 | 138.542 | 1.53952 | 0.150 |
|  | GO:0021539 | subthalamus development | 1 | 138.542 | 1.53952 | 0.150 |
|  | GO:0021548 | pons development | 1 | 138.542 | 1.53952 | 0.150 |
|  | GO:0021750 | vestibular nucleus development | 1 | 138.542 | 1.53952 | 0.150 |
|  | GO:0021771 | lateral geniculate nucleus development | 1 | 138.542 | 1.53952 | 0.150 |
|  | GO:0021920 | regulation of transcription from RNA polymerase II promoter involved in spinal cord association neuron specification | 1 | 138.542 | 1.53952 | 0.150 |
|  | GO:0021935 | cerebellar granule cell precursor tangential migration | 1 | 138.542 | 1.53952 | 0.150 |
|  | GO:0030865 | cortical cytoskeleton organization | 1 | 138.542 | 1.53952 | 0.150 |
|  | GO:0031645 | negative regulation of neurological system process | 1 | 138.542 | 1.53952 | 0.150 |
|  | GO:0032225 | regulation of synaptic transmission, dopaminergic | 1 | 138.542 | 1.53952 | 0.150 |
|  | GO:0032328 | alanine transport | 1 | 138.542 | 1.53952 | 0.150 |
|  | GO:0034230 | enkephalin processing | 1 | 138.542 | 1.53952 | 0.150 |
|  | GO:0034231 | islet amyloid polypeptide processing | 1 | 138.542 | 1.53952 | 0.150 |
|  | GO:0035883 | enteroendocrine cell differentiation | 1 | 138.542 | 1.53952 | 0.150 |
|  | GO:0042137 | sequestering of neurotransmitter | 1 | 138.542 | 1.53952 | 0.150 |
|  | GO:0042182 | ketone catabolic process | 1 | 138.542 | 1.53952 | 0.150 |
|  | GO:0042320 | regulation of circadian sleep/wake cycle, REM sleep | 1 | 138.542 | 1.53952 | 0.150 |
|  | GO:0043134 | regulation of hindgut contraction | 1 | 138.542 | 1.53952 | 0.150 |
|  | GO:0045299 | otolith mineralization | 1 | 138.542 | 1.53952 | 0.150 |
|  | GO:0045992 | negative regulation of embryonic development | 1 | 138.542 | 1.53952 | 0.150 |
|  | GO:0046588 | negative regulation of calcium-dependent cell-cell adhesion | 1 | 138.542 | 1.53952 | 0.150 |
|  | GO:0048692 | negative regulation of axon extension involved in regeneration | 1 | 138.542 | 1.53952 | 0.150 |
|  | GO:0051355 | proprioception involved in equilibrioception | 1 | 138.542 | 1.53952 | 0.150 |
|  | GO:0051589 | negative regulation of neurotransmitter transport | 1 | 138.542 | 1.53952 | 0.150 |
|  | GO:0051977 | lysophospholipid transport | 1 | 138.542 | 1.53952 | 0.150 |
|  | GO:0060078 | regulation of postsynaptic membrane potential | 1 | 138.542 | 1.53952 | 0.150 |
|  | GO:0060165 | regulation of timing of subpallium neuron differentiation | 1 | 138.542 | 1.53952 | 0.150 |
|  | GO:0060404 | axonemal microtubule depolymerization | 1 | 138.542 | 1.53952 | 0.150 |
|  | GO:0060580 | ventral spinal cord interneuron fate determination | 1 | 138.542 | 1.53952 | 0.150 |
|  | GO:0060730 | regulation of intestinal epithelial structure maintenance | 1 | 138.542 | 1.53952 | 0.150 |
|  | GO:0061100 | lung neuroendocrine cell differentiation | 1 | 138.542 | 1.53952 | 0.150 |
|  | GO:0061102 | stomach neuroendocrine cell differentiation | 1 | 138.542 | 1.53952 | 0.150 |
|  | GO:0061103 | carotid body glomus cell differentiation | 1 | 138.542 | 1.53952 | 0.150 |
|  | GO:0061104 | adrenal chromaffin cell differentiation | 1 | 138.542 | 1.53952 | 0.150 |
|  | GO:0070445 | regulation of oligodendrocyte progenitor proliferation | 1 | 138.542 | 1.53952 | 0.150 |
|  | GO:0070462 | plus-end specific microtubule depolymerization | 1 | 138.542 | 1.53952 | 0.150 |
|  | GO:0070684 | seminal clot liquefaction | 1 | 138.542 | 1.53952 | 0.150 |
|  | GO:0071242 | cellular response to ammonium ion | 1 | 138.542 | 1.53952 | 0.150 |
|  | GO:0071259 | cellular response to magnetism | 1 | 138.542 | 1.53952 | 0.150 |
|  | GO:0071622 | regulation of granulocyte chemotaxis | 1 | 138.542 | 1.53952 | 0.150 |
|  | GO:0090104 | pancreatic epsilon cell differentiation | 1 | 138.542 | 1.53952 | 0.150 |
|  | GO:0090274 | positive regulation of somatostatin secretion | 1 | 138.542 | 1.53952 | 0.150 |
|  | GO:1902004 | positive regulation of beta-amyloid formation | 1 | 138.542 | 1.53952 | 0.150 |
|  | GO:0006614 | SRP-dependent cotranslational protein targeting to membrane | 4 | 5.278 | 1.539515 | 0.150 |
|  | GO:0006836 | neurotransmitter transport | 2 | 15.394 | 1.530624 | 0.151 |
|  | GO:0010765 | positive regulation of sodium ion transport | 2 | 15.394 | 1.530624 | 0.151 |
|  | GO:0030041 | actin filament polymerization | 2 | 15.394 | 1.530624 | 0.151 |
|  | GO:0007286 | spermatid development | 3 | 7.557 | 1.527437 | 0.151 |
|  | GO:0042127 | regulation of cell proliferation | 4 | 5.131 | 1.497163 | 0.162 |
|  | GO:0007202 | activation of phospholipase C activity | 3 | 7.292 | 1.484637 | 0.166 |
|  | GO:0001539 | ciliary or flagellar motility | 2 | 13.854 | 1.440691 | 0.178 |
|  | GO:0043278 | response to morphine | 2 | 13.854 | 1.440691 | 0.178 |
|  | GO:0050850 | positive regulation of calcium-mediated signaling | 2 | 13.854 | 1.440691 | 0.178 |
|  | GO:0051412 | response to corticosterone stimulus | 2 | 13.854 | 1.440691 | 0.178 |
|  | GO:0051965 | positive regulation of synapse assembly | 2 | 13.854 | 1.440691 | 0.178 |
|  | GO:0090398 | cellular senescence | 2 | 13.854 | 1.440691 | 0.178 |
|  | GO:0008217 | regulation of blood pressure | 3 | 6.927 | 1.423516 | 0.185 |
|  | GO:0014003 | oligodendrocyte development | 2 | 13.194 | 1.399288 | 0.193 |
|  | GO:0042220 | response to cocaine | 2 | 13.194 | 1.399288 | 0.193 |
|  | GO:0045907 | positive regulation of vasoconstriction | 2 | 13.194 | 1.399288 | 0.193 |
|  | GO:0008156 | negative regulation of DNA replication | 2 | 12.595 | 1.359957 | 0.209 |
|  | GO:0035094 | response to nicotine | 2 | 12.595 | 1.359957 | 0.209 |
|  | GO:0043410 | positive regulation of MAPK cascade | 3 | 6.494 | 1.347202 | 0.213 |
|  | GO:0007173 | epidermal growth factor receptor signaling pathway | 5 | 3.744 | 1.34193 | 0.213 |
|  | GO:0008089 | anterograde axon cargo transport | 2 | 12.047 | 1.322509 | 0.213 |
|  | GO:0010043 | response to zinc ion | 2 | 12.047 | 1.322509 | 0.213 |
|  | GO:0043542 | endothelial cell migration | 2 | 12.047 | 1.322509 | 0.213 |
|  | GO:0045909 | positive regulation of vasodilation | 2 | 12.047 | 1.322509 | 0.213 |
|  | GO:0070373 | negative regulation of ERK1 and ERK2 cascade | 2 | 12.047 | 1.322509 | 0.213 |
|  | GO:0071310 | cellular response to organic substance | 2 | 12.047 | 1.322509 | 0.213 |
| **KEGG Pathway** | 04080 | Neuroactive ligand-receptor interaction | 18 | 9.003 | 10.927 | 0.000 |
|  | 04724 | Glutamatergic synapse | 12 | 14.583 | 9.709 | 0.000 |
|  | 05012 | Parkinson's disease | 11 | 10.732 | 7.477 | 0.000 |
|  | 04932 | Non-alcoholic fatty liver disease (NAFLD) | 11 | 10.092 | 7.198 | 0.000 |
|  | 04721 | Synaptic vesicle cycle | 8 | 17.593 | 7.121 | 0.000 |
|  | 05010 | Alzheimer's disease | 11 | 9.071 | 6.719 | 0.000 |
|  | 00190 | Oxidative phosphorylation | 10 | 10.417 | 6.674 | 0.000 |
|  | 04723 | Retrograde endocannabinoid signaling | 9 | 12.345 | 6.643 | 0.000 |
|  | 05016 | Huntington's disease | 11 | 7.896 | 6.107 | 0.000 |
|  | 05033 | Nicotine addiction | 6 | 20.781 | 5.769 | 0.000 |
|  | 04020 | Calcium signaling pathway | 10 | 7.740 | 5.472 | 0.000 |
|  | 04950 | Maturity onset diabetes of the young | 5 | 26.643 | 5.352 | 0.000 |
|  | 04260 | Cardiac muscle contraction | 7 | 12.433 | 5.180 | 0.000 |
|  | 04911 | Insulin secretion | 7 | 11.409 | 4.927 | 0.000 |
|  | 04727 | GABAergic synapse | 7 | 11.020 | 4.826 | 0.000 |
|  | 04971 | Gastric acid secretion | 6 | 11.233 | 4.175 | 0.001 |
|  | 04726 | Serotonergic synapse | 7 | 8.659 | 4.134 | 0.001 |
|  | 04022 | cGMP-PKG signaling pathway | 8 | 6.637 | 3.890 | 0.001 |
|  | 00650 | Butanoate metabolism | 4 | 20.525 | 3.783 | 0.001 |
|  | 04728 | Dopaminergic synapse | 7 | 7.518 | 3.739 | 0.002 |
|  | 04923 | Regulation of lipolysis in adipocytes | 5 | 12.370 | 3.665 | 0.002 |
|  | 05032 | Morphine addiction | 6 | 9.135 | 3.663 | 0.002 |
|  | 04713 | Circadian entrainment | 6 | 8.750 | 3.559 | 0.002 |
|  | 04024 | cAMP signaling pathway | 8 | 5.570 | 3.356 | 0.003 |
|  | 01100 | Metabolic pathways | 22 | 2.470 | 3.332 | 0.003 |
|  | 04720 | Long-term potentiation | 5 | 10.496 | 3.322 | 0.003 |
|  | 04725 | Cholinergic synapse | 6 | 7.489 | 3.185 | 0.004 |
|  | 04918 | Thyroid hormone synthesis | 5 | 9.756 | 3.171 | 0.004 |
|  | 05030 | Cocaine addiction | 4 | 11.310 | 2.756 | 0.010 |
|  | 04970 | Salivary secretion | 5 | 7.783 | 2.714 | 0.011 |
|  | 04972 | Pancreatic secretion | 5 | 7.216 | 2.564 | 0.015 |
|  | 04261 | Adrenergic signaling in cardiomyocytes | 6 | 5.617 | 2.517 | 0.016 |
|  | 04921 | Oxytocin signaling pathway | 6 | 5.261 | 2.370 | 0.022 |
|  | 05031 | Amphetamine addiction | 4 | 8.271 | 2.242 | 0.028 |
|  | 04270 | Vascular smooth muscle contraction | 5 | 5.773 | 2.131 | 0.035 |
|  | 00072 | Synthesis and degradation of ketone bodies | 2 | 27.708 | 2.046 | 0.042 |
|  | 04360 | Axon guidance | 5 | 5.454 | 2.023 | 0.043 |
|  | 04914 | Progesterone-mediated oocyte maturation | 4 | 6.370 | 1.828 | 0.064 |
|  | 04940 | Type I diabetes mellitus | 3 | 9.666 | 1.827 | 0.064 |
|  | 04912 | GnRH signaling pathway | 4 | 6.090 | 1.758 | 0.073 |
|  | 00280 | "Valine, leucine and isoleucine degradation" | 3 | 8.843 | 1.718 | 0.078 |
|  | 04750 | Inflammatory mediator regulation of TRP channels | 4 | 5.655 | 1.644 | 0.088 |
|  | 04913 | Ovarian steroidogenesis | 3 | 8.313 | 1.643 | 0.088 |
|  | 04742 | Taste transduction | 3 | 7.993 | 1.595 | 0.096 |
|  | 04730 | Long-term depression | 3 | 6.927 | 1.424 | 0.140 |
|  | 04144 | Endocytosis | 6 | 3.222 | 1.335 | 0.168 |
|  | 04964 | Proximal tubule bicarbonate reclamation | 2 | 12.047 | 1.323 | 0.169 |
|  | 04080 | Neuroactive ligand-receptor interaction | 18 | 9.003 | 10.927 | 0.000 |
|  | 04724 | Glutamatergic synapse | 12 | 14.583 | 9.709 | 0.000 |
